# Effect of histology on the efficacy of first-line immune checkpoint inhibitors in advanced non-small cell lung cancer: a systematic review and network meta-analysis

**DOI:** 10.3389/fimmu.2026.1850384

**Published:** 2026-06-03

**Authors:** Sihan Li, Tianyu He, Jun Chen, Tingting Liu, Xinyu Zhao, Jun Dang

**Affiliations:** 1 Department of Radiation Oncology, The First Hospital of China Medical University, Shenyang, China; 2 Department of Radiation Oncology, Shenyang Tenth People’s Hospital, Shenyang, China; 3 Department of Radiation Oncology, Anshan Cancer Hospital, Anshan, China

**Keywords:** immune checkpoint inhibitor, network meta-analysis, non-small cell lung cancer, non-squamous, squamous

## Abstract

**Background:**

First-line immune checkpoint inhibitors (ICIs) have become the standard of care for advanced non-small cell lung cancer (NSCLC). However, whether tumor histology influences the efficacy of ICIs remains unclear.

**Methods:**

We systematically searched PubMed, Embase, the Cochrane Library, Web of Science, and Scopus for randomized controlled trials (RCTs) published up to February 28, 2026. Eligible studies compared ICIs with chemotherapy, or two ICI-based regimens, as first-line treatment for advanced squamous (SQ) or non-squamous (non-SQ) NSCLC. Trials that enrolled patients with NSCLC and reported outcomes stratified by histology were also eligible. The primary outcome was overall survival (OS), reported as hazard ratios (HRs) with 95% credible intervals. A fixed-effects consistency model was used for the statistical analysis.

**Results:**

Forty-one phase 3 RCTs comprising 23, 871 patients and 31 ICI-based regimens were included. The efficacy of individual ICI regimens varied by histological subtype. For example, toripalimab plus chemotherapy was associated with superior OS compared with most other ICI regimens (HR range: 0.42–0.65) and ranked as the best treatment in non-SQ-NSCLC (surface under the cumulative ranking curve [SUCRA] = 0.97). In contrast, the same regimen showed inferior OS relative to many comparators (HR range for comparators vs. toripalimab plus chemotherapy: 0.47–0.65) and had the lowest OS ranking in SQ-NSCLC (SUCRA = 0.09). In the PD-L1 < 1% subgroup, nivolumab plus ipilimumab demonstrated a trend toward better OS compared with pembrolizumab plus chemotherapy (HR = 0.59) and ranked as the best regimen for SQ-NSCLC (SUCRA = 0.83), whereas pembrolizumab plus chemotherapy provided the greatest OS benefit for non-SQ-NSCLC (SUCRA = 0.90). In the PD-L1 ≥ 50% subgroup, atezolizumab plus chemotherapy ranked second for OS benefit in SQ-NSCLC but was the least effective combination in non-SQ-NSCLC; conversely, cemiplimab plus chemotherapy was the least effective combination in SQ-NSCLC but ranked second in non-SQ-NSCLC.

**Conclusions:**

The efficacy of individual first-line ICI regimens appear to vary by histological subtype across PD-L1 expression levels. These findings suggest that PD-L1 status alone might not be sufficient to guide treatment selection, and that histological subtype could be considered in clinical decision-making for advanced NSCLC.

## 1 Introduction

Lung cancer remains the leading cause of cancer-related mortality globally, with non-small cell lung cancer (NSCLC) accounting for approximately 85% of all cases (1). NSCLC is broadly classified into squamous cell carcinoma (SQ-NSCLC) and non-squamous cell carcinoma (non-SQ-NSCLC), the latter of which is predominantly adenocarcinoma. Compared with adenocarcinoma, squamous histology is characterized by a distinct molecular profile, marked by a substantially lower prevalence of targetable oncogenic drivers such as *EGFR* mutations and *ALK* rearrangements (2), thereby limiting the applicability of targeted therapies. For decades, platinum-based chemotherapy served as the standard first-line treatment for advanced SQ-NSCLC; however, its clinical benefit remained modest, with poor long-term survival outcomes (3).

The advent of immune checkpoint inhibitors (ICIs) targeting PD-1, PD-L1, or CTLA-4 has profoundly reshaped the therapeutic landscape for advanced NSCLC. A series of phase 3 randomized controlled trials (RCTs) (4–53) have consistently demonstrated that first-line ICI-based regimens, whether as monotherapy or in combination, confer superior efficacy over chemotherapy alone. However, the clinical benefit of ICIs is not uniform across histological subtypes. For instance, in the CHOICE-01 trial (38), toripalimab plus chemotherapy significantly improved overall survival (OS) in patients with non-SQ-NSCLC (hazard ratio [HR] = 0.49, 95% confidence interval [CI]: 0.35–0.69), but not in those with SQ-NSCLC (HR = 1.09, 95% CI: 0.77–1.56). Conversely, in the PEARL trial (30), durvalumab achieved superior OS over chemotherapy in patients with SQ-NSCLC (HR = 0.75, 95% CI: 0.58–0.98), whereas no significant benefit was observed in non-SQ-NSCLC (HR = 0.91, 95% CI: 0.73–1.13). SQ-NSCLC and non-SQ-NSCLC are biologically distinct entities, differing in tumor mutational burden, immune contexture, and tumor microenvironment composition (54–56). Moreover, SQ-NSCLC is more commonly associated with a heavier smoking history and a greater burden of comorbidities (2). These divergent biological and clinical features may collectively modulate responsiveness to ICI therapy.

To date, multiple ICI agents have been approved for the treatment of NSCLC by the U.S. Food and Drug Administration (FDA) and/or the Chinese National Medical Products Administration (NMPA). However, head-to-head RCTs directly comparing the efficacy of ICIs between SQ-NSCLC and non-SQ-NSCLC are lacking. The precise impact of histology on the survival benefits conferred by ICIs, particularly in the first-line setting, remains to be quantitatively characterized. To address this gap, we conducted a network meta-analysis evaluating the comparative efficacy of first-line ICI-based regimens separately in patients with advanced SQ-NSCLC and non-SQ-NSCLC, aiming to provide histology-specific evidence to support clinical decision-making.

## 2 Materials and methods

### 2.1 Literature search and inclusion criteria

This meta-analysis was conducted in accordance with the Preferred Reporting Items for Systematic Reviews and Meta-analyses (PRISMA) guidelines (57). Two authors (S.L. and T.H.) independently searched PubMed, Embase, the Cochrane Library, Web of Science, and Scopus for eligible RCTs published up to February 28, 2026. The detailed search strategy is provided in **Supplementary Table 1**. Abstracts from recent major meetings (the American Society of Clinical Oncology, European Society for Medical Oncology, European Lung Cancer Congress, and World Conference on Lung Cancer) were also reviewed. RCTs were included if they met the following criteria: (1) phase 3 trials comparing first-line ICI-based regimens (monotherapy or combination) with chemotherapy, or comparing two ICI-based regimens, in patients with advanced SQ-NSCLC or non-SQ-NSCLC; and (2) availability of overall survival (OS) and/or progression-free survival (PFS) data, and/or grade 3–5 treatment-related adverse events (TRAEs). Trials that enrolled NSCLC patients and reported survival outcomes stratified by histology were also eligible. When multiple publications originated from the same trial, the most recent report with the longest follow-up was included.

### 2.2 Data extraction and risk of bias assessment

Two authors (S.L. and T.H.) independently extracted the following information from each eligible study: (1) trial characteristics: trial name, geographic region, sample size, median follow-up duration, and treatment regimen; (2) patient characteristics: age, sex, Eastern Cooperative Oncology Group (ECOG) performance status, smoking history, and PD-L1 expression level; and (3) outcome data: hazard ratios (HRs) with corresponding 95% confidence intervals (CIs) for OS and PFS, and odds ratios (ORs) with 95% CIs for grade 3–5 TRAEs. The risk of bias of the included trials was assessed using the Cochrane Risk of Bias Tool (58).

### 2.3 Statistical analysis

The primary outcome was OS; secondary outcomes were PFS and grade 3–5 TRAEs. A Bayesian network meta-analysis was performed using the Markov chain Monte Carlo simulation method (59), as implemented in R software (version 4.3.2) with the gemtc package. Given that most direct comparisons were informed by a single trial, a fixed-effects consistency model was applied (60). Treatment effects were estimated using HRs or ORs with their 95% credible intervals (CrIs). For each outcome, four independent Markov chains were run simultaneously, with 10, 000 burn-in iterations followed by 150, 000 sample iterations per chain, using a step size of 10 iterations to obtain the posterior distribution. Trace plots and the Brooks–Gelman–Rubin diagnostic were used to assess model convergence (61). The surface under the cumulative ranking curve (SUCRA) (62) was calculated to rank the probability of each treatment being most effective or least toxic, with a SUCRA value of 1 indicating the best and 0 the worst treatment.

Global inconsistency was evaluated by comparing the goodness-of-fit between consistency and inconsistency models using the deviance information criterion (DIC) (63, 64); a lower DIC value indicated better fit and absence of significant global inconsistency. Local inconsistency between direct and indirect evidence was assessed by comparing effect estimates derived from pairwise and network meta-analyses (63, 64). The node-splitting method (63, 65) was used to evaluate inconsistency within treatment loops, with a two-sided *P* value < 0.05 indicating statistically significant inconsistency. Heterogeneity was assessed using the Q and I² tests for comparisons informed by two or more trials. Subgroup analyses were performed according to PD-L1 expression level. Sensitivity analysis was conducted by using a random-effects mode or omitting mixed-histology trials.

## 3 Results

### 3.1 Study selection and characteristics

**Figure 1:**
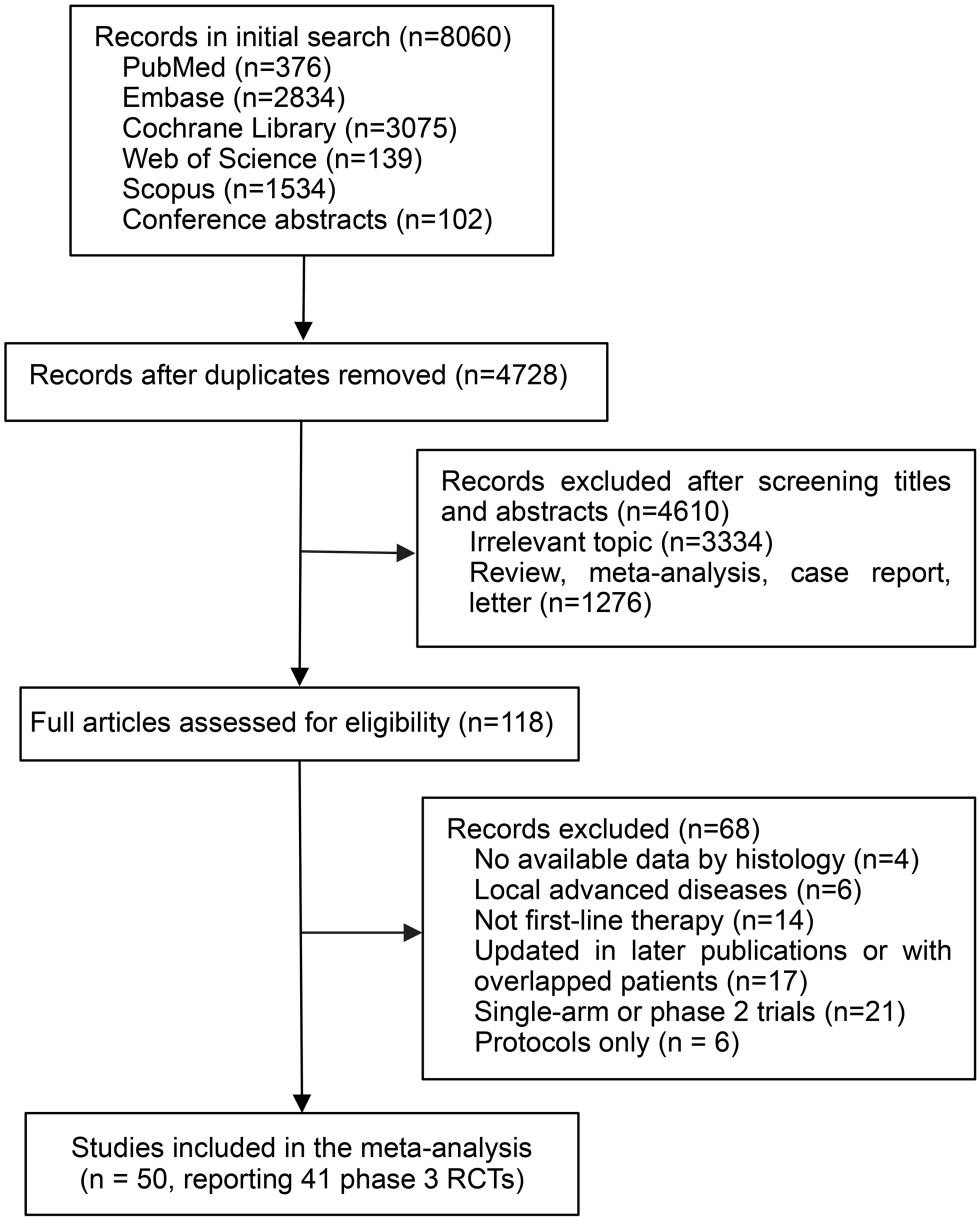
Literature search and selection. RCTs, randomized controlled trials.

The initial literature search yielded 8, 060 records. After screening titles and abstracts, 7, 942 articles were excluded. The remaining 118 articles were retrieved for full-text assessment. Ultimately, 50 publications (4–53), reporting data from 41 phase 3 RCTs involving 23, 871 patients (9, 724 with SQ-NSCLC and 14, 147 with non-SQ-NSCLC) and 31 ICI-based treatment regimens, were included in the meta-analysis. The study selection process is summarized in **Figure 1**. Among the 41 phase 3 RCTs, 9 were conducted exclusively in patients with SQ-NSCLC, 9 exclusively in patients with non-SQ-NSCLC, and 23 enrolled both histologies and reported OS and/or PFS data for the SQ-NSCLC and non-SQ-NSCLC subgroups. Thirty-four trials compared ICIs (monotherapy or combination) with chemotherapy ± bevacizumab, and 7 compared two ICI-based regimens. Seventeen RCTs were conducted in Asian countries, and the remaining 24 were multinational. Most patients had an ECOG performance status of 0–1 and were negative for *EGFR* mutations and *ALK* rearrangements. The median follow-up duration across trials was 25.2 months (interquartile range [IQR], 15.1–38.0). The main characteristics and survival outcomes of each trial are summarized in **Table 1**.

**Table 1:** Main characteristics and outcomes of included trials.

Trial name/year	Region	Median follow-up (m)	Treatment (sample size: SQ; non-SQ)	Histological type	Median age	Males (n, %)	Asian (n, %)	Never smoker (%)	PD-L1 <1%/≥1%/≥50% (%) (E;C)	HR for OS (95% CI) (SQ; non-SQ)	HR for PFS(95% CI) (SQ; non-SQ)
CheckMate-026/2017 (4)	Muti	13.5	Nivolumab/CT (65/64;206/206)	NSCLC	63/65	68/55	11/6	11/11	0/100/33; 0/100/45	0.82(0.54-1.24); 1.17(0.91-1.52)	0.83(0.54-1.26); 1.29(1.02-1.63)
CheckMate-227/2019 (5)	Multi	≥11.2	Nivolumab+ipilimumab/CT(163/162;419/421)	NSCLC	64/64	67/66	21/21	14/13	32/68/35; 32/68/33	0.62(0.49-0.80); 0.79(0.67-0.93)	NR
CheckMate-227(part2)/2023 (6)	Multi	36.0	Nivolumab+CT/CT (107/105;270/273)	NSCLC	63/64	70/70	24/24	15/20	43/57/26; 43/57/22	0.70(0.51-0.94); 0.82(0.68-1.01)	0.51(0.37-0.70); 0.67(0.55-0.82)
CheckMate-9LA/2021 (7)/2025 (8)	Multi	75.8	Nivolumab+ipilimumab+ CT/CT(115/112;246/246)	NSCLC	65/65	70/70	8/8	13/14	40/60/22; 39/61/29	0.65(0.49-0.85); 0.79(0.65-0.96)	0.57(0.42-0.78); 0.74(0.60-0.92)
TASUKI-52/2025 (9)	Multi	13.7	Nivolumab+Bev+CT/Bev+CT(275/275)	non-SQ- NSCLC	66/66	75/75	100	22/20	44/56/27; 44/56/27	0.71(0.57-0.88)	0.59(0.47-0.73)
Keynote-024/2016 (10)/2019 (11)	Muti	25.2	Pembrolizumab/CT (29/27;125/124)	NSCLC	65/66	60/63	NR	3/13	0/100/100; 0/100/100	0.73(0.38-1.39); 0.58(0.41-0.83)	0.35(0.17-0.71); 0.55(0.39-0.76)
Keynote-042/2019 (12)	Muti	12.8	Pembrolizumab/CT (243/249;394/388)	NSCLC	63/63	71/71	29/29	22/22	0/100/47; 0/100/47	0.75(0.60-0.93; 0.86(0.72-1.03))	NR
Keynote-189/2023 (13)	Multi	64.6	Pembrolizumab+CT/CT(410/206)	non-SQ- NSCLC	65/64	62/53	1/3	12/12	31/63/32; 31/62/34	0.60(0.50-0.72)	0.50(0.42-0.60)
Keynote-407/2023 (14)	Multi	56.9	Pembrolizumab+CT/CT(278/281)	SQ-NSCLC	65/65	79/84	19/19	8/7	34/63/26; 35/63/26	0.71(0.59-0.85)	0.62(0.52-0.74)
IMpower-110/2021 (15)	Multi	31.3	Atezolizumab/CT (27/23;80/75)	NSCLC	63/66	74/65	19/15	8/15	0/100/100; 0/100/100	0.91(0.45-1.83); 0.72(0.48-1.08)	NR
IMpower130/2019 (16)	Multi	18.5	Atezolizumab+CT/CT(451/228)	non-SQ- NSCLC	64/65	59/59	3/1	11/7	52/48/20; 53/47/19	0.79(0.64-0.98)	0.64(0.54-0.77)
IMpower-131/2020 (17)	Multi	26.8	Atezolizumab+CT/CT(343/340)	SQ-NSCLC	65/65	82/82	12/11	9/7	47/53/14; 50/50/13	0.88(0.73-1.05)	0.71(0.60-0.85)
IMpower132/2021 (18)	Multi	14.8	Atezolizumab+CT/CT(292/286)	non-SQ- NSCLC	64/63	66/67	24/23	13/11	50/50/14; 45/55/12	0.86(0.71-1.06)	0.60(0.49-0.72)
IMpower150/2018 (19)/2021 (20)	Multi	39.8	Atezolizumab+Bev+CT/Bev+CT(359/338)	non-SQ-NSCLC	63/63	60/60	14/12	21/19	45/55/24; 50/50/23	0.80(0.67-0.95)	0.62(0.52-0.74)
			Atezolizumab+CT/Bev+CT(350/338)	non-SQ- NSCLC	63/63	60/60	12/12	19/19	41/59/24; 50/50/23	0.84(0.71-1.00)	NR
IPSOS/2023 (21)	Multi	41.0	Atezolizumab/CT (129/64;173/87)	NSCLC	75/75	73/72	25/25	12/13	50/42/17; 40/52/17	0.80(0.58-1.12); 0.77(0.58-1.03)	NR
BFAST/2022 (22)	Muti	18.2	Atezolizumab/CT (33/34;113/112)	NSCLC	65/66	73/74	14/22	3/1	0/100/NR; 0/100/NR	1.22(0.67-2.21); 0.78(0.55-1.11)	1.14(0.68-1.92); 0.65(0.48-0.88)
EMPOWER-Lung1/2025 (23)	Multi	59.6	Cemiplimab/CT (123/122;161/159)	NSCLC	63/64	88/83	11/10	0/0	0/100/100; 0/100/100	0.51(0.38-0.69); 0.66(0.50-0.88)	0.44(0.32-0.60); 0.55(0.42-0.72)
EMPOWER-Lung3/2023 (24)2025 (25)	Multi	28.4	Cemiplimab+CT/CT(133/67;179/87)	NSCLC	63/63	86/80	14/10	14/16	30/70/33; 29/71/32	0.68(0.49-0.94); 0.62(0.46-0.82)	0.56(0.41-0.78); 0.53(0.39-0.71)
JAVELIN Lung 100/2024 (26)	Multi	NR	Avelumab/CT (47/66;104/150)	NSCLC	64/63	74/73	25/22	11/14	0/100/100; 0/100/100	0.94(0.61-1.45); 0.81(0.60-1.10)	0.63(0.38-1.05); 0.74(0.54-1.02)
MYSTIC/2020 (27)	Muti	30.2	Durvalumab/CT (52/52;111/110)	NSCLC	64/65	69/65	36/29	15/13	0/100/NR; 0/100/NR	0.89(0.57-1.37); 0.70(0.51-0.96)	NR
POSEIDON/2025 (28)	Multi	63.4	Durvalumab+CT/CT(128/122;209/214)	NSCLC	65/64	75/74	36/38	25/23	33/66/28; 39/61/29	0.82(0.64-1.07); 0.81(0.66-1.00)	NR
			Durvalumab+ tremelimumab+CT/CT(124/122;214/214)	NSCLC	63/64	80/74	29/38	18/23	37/63/30; 39/61/29	0.85(0.65-1.10; 0.69(0.56-0.85)	NR
NEPTUNE/2023 (29)	Multi	32.9	Durvalumab+ Tremelimumab/CT(166/170;244/243)	NSCLC	63/65	72/74	21/24	18/18	22/78/NR; 25/75/NR	0.87(0.69-1.10); 1.13(0.92-1.39)	NR
PEARL/2025 (30)	Multi	53.7	Durvalumab/CT (132/133;203/201)	NSCLC	61/64	80/81	78/82	21/22	0/100/74; 0/100/74	0.75(0.58-0.98); 0.91(0.73-1.13)	NR
RATIONALE 304/2024 (31)	China	19.3	Tislelizumab+CT/CT(223/111)	non-SQ- NSCLC	60/61	75/71	100	34/41	41/57/33; 43/57/32	0.89(0.63-1.27)	0.63(0.47-0.86)
RATIONALE-307/2024 (32)	China	20.5	Tislelizumab+PC/CT (120/121)	SQ-NSCLC	60/62	89/92	100	20/19	39/60/35; 37/60/34	0.69(0.50-0.95)	0.45(0.33-0.62)
			Tislelizumab+nPC/CT (119/121)	SQ-NSCLC	63/62	94/92	100	10/19	39/60/35; 37/60/34	0.84(0.61-1.14)	0.43(0.31-0.60)
CameL/2024 (33)	China	65.2	Camrelizumab+CT/CT(205/207)	non-SQ- NSCLC	59/61	71/72	100	38/37	24/67/15; 33/57/10	0.74(0.58-0.93)	0.55(0.44-0.68)
CameL-sq/2022 (34)	China	13.5	Camrelizumab+CT/CT(193/196)	SQ-NSCLC	64/62	93/92	100	11/12	47/49/19; 49/47/22	0.55(0.40-0.75)	0.37(0.29-0.47)
GEMSTONE-302/2022 (35)/2025 (36)	China	43.5	Sugemalimab+CT/CT(129/63;191/96)	NSCLC	62/64	79/81	100	27/25	39/61/NR; 40/60/NR	0.61(0.43-0.87); 0.72(0.53-0.98)	0.34(0.24-0.48); 0.59(0.45-0.79)
CHOICE-01/2023 (37)/2024 (38)	China	21.2	Toripalimab+CT/CT(147/73;162/83)	NSCLC	63/61	80/83	100	31/31	35/65/NR; 34/66/NR	1.09(0.77-1.56); 0.49(0.35-0.69)	0.49(0.35-0.69); 0.48(0.35-0.66)
ASTRUM-004/2024 (39)	China	31	Serplulimab+CT/CT(358/179)	SQ-NSCLC	63/63	90/93	67/67	14/11	38/62/29; 38/62/30	0.73(0.58-0.93)	0.53(0.42-0.67)
ASTRUM-002/2025 (40)	China	23.4	Serplulimab+CT/CT(214/240)	non-SQ- NSCLC	62/61	73/74	100	33/33	39/59/29; 32/65/30	NR	0.55(0.43-0.69)
ORIENT-11/2021 (41)/2022 (42)	China	30.8	Sintilimab+CT/CT(266/131)	non-SQ- NSCLC	61/61	77/76	100	36/34	32/68/40; 34/66/47	0.65(0.50-0.85)	0.49(0.38-0.63)
ORIENT-12/2021 (43)	China	12.9	Sintilimab+CT/CT(179/178)	SQ-NSCLC	64/62	91/92	100	13/17	33/67/32; 35/65/35	0.57(0.35-0.91)	0.54(0.42-0.68)
AK105-302/2024 (44)	China	24.7	Penpulimab+CT/CT(175/175)	SQ-NSCLC	61/62	93/93	100	11/13	34/66/18; 33/67/18	0.55(0.40-0.75)	0.43(0.33-0.56)
Study-104/2017 (45)	Muti	12.5	Ipilimumab+CT/CT(388/361)	SQ-NSCLC	64/64	84/85	27/30	NR	NR	0.91(0.77-1.07)	0.87(0.75-1.01)
Pooled analysis of CheckMate 227-9LA/2025 (46)	Muti	73.7	Nivolumab+ipilimumab ± CT/CT(82/82;240/233)	NSCLC	64/64	73/69	NR	13/14	100/0/0; 100/0/0	0.51(0.36-0.72); 0.69(0.57-0.84)	0.60(0.43-0.85); 0.77(0.63-0.95)
KEYNOTE-598/2021 (47)	Muti	20.6	Pembrolizumab+ipilimumab/Pembrolizumab (77/81;207/203)	NSCLC	64/65	71/67	11/11	10/9	0/100/100; 0/100/100	1.16(0.76-1.78); 1.04(0.78-1.38)	0.98(0.68-1.42); 1.12(0.88-1.43)
APPLE/2024 (48)	Japan	NR	Atezolizumab+Bev+CT/Atezolizumab+CT(205/206)	non-SQ- NSCLC	67/68	68/64	100	28/28	35/49/20; 34/43/20	0.86(0.65-1.13)	0.86(0.70-1.07)
NIPPON/2024 (49)	Japan	15.3	Pembrolizumab+CT/Nivolumab+ipilimumab +CT(32/33;115/115)	NSCLC	68/68	79/81	100	12/11	41/49/17; 39/49/16	1.02(0.47-2.21); 0.94(0.62-1.42)	1.18(0.65-2.12); 0.90(0.66-1.22)
TQB2450-III-12/2025 (50)	China	7.0	Benmelstobart+CT/Tislelizumab+CT (278/284)	SQ-NSCLC	65/64	90/92	100	17/15	37/63/17; 38/62/18	NR	0.64(0.45-0.93)
CAMPASS/2025 (51)	China	11.4	Benmelstobart+anlotinib/Pembrolizumab (213/107;141/70)	NSCLC	65/65	85/84	100	25/19	0/100/45; 0/100/45	NR	0.63(0.46-0.86); 0.83(0.54-1.27)
HARMONi-2/2025 (52)	China	8.7	Ivonescimab/pembrolizumab (90/91;108/109)	NSCLC	65/66	83/85	100	20/19	0/100/42; 0/100/43	NR	0.50(0.33-0.76); 0.55(0.36-0.84)
HARMONi-6/2025 (53)	China	10.4	Ivonescimab+CT/Tislelizumab+CT (266/266)	SQ-NSCLC	64/64	96/89	100	8/14	39/61/18; 39/6123	NR	0.60(0.46-0.78)

OS, overall survival; PFS, progression-free survival; HR, hazard ratio; CI, confidence interval; SQ, squamous cell carcinoma; NSCLC, non-small cell lung cancer; CT, chemotherapy; Bev, bevacizumab; PC, paclitaxel plus carboplatin; nPC, nab-paclitaxel plus carboplatin; E, experimental group; C, control group; NR, not reported.

### 3.2 Network meta-analysis

**Figure 2:**
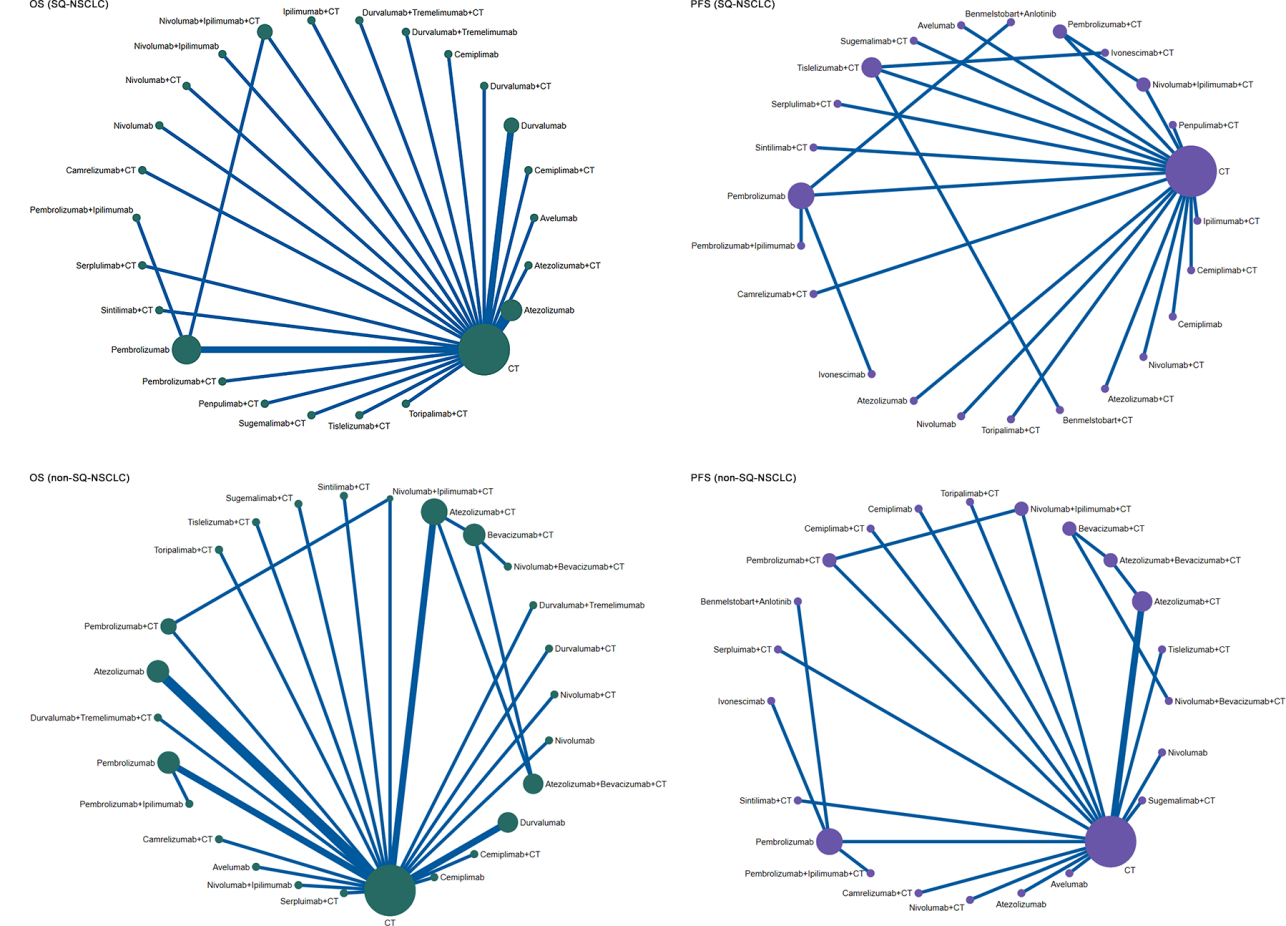
Eligible comparisons in the network meta-analysis. OS, overall survival; PFS, progression-free survival; SQ-NSCLC, squamous non-small cell lung cancer; Anl, anlotinib; Ate, atezolizumab; Ave, avelumab; Ben, benmelstobart; Bev, bevacizumab; Cam, camrelizumab; Cem, cemiplimab; Dur, durvalumab; Ipi, ipilimumab; Ivo, ivonescimab; Niv, nivolumab; Pem, pembrolizumab; Pen, penpulimab; Ser, serplulimab; Sin, sintilimab; Sug, sugemalimab; Tis, tislelizumab; Tor, toripalimab; Tre, tremelimumab; CT, chemotherapy.

**Figure 3:**
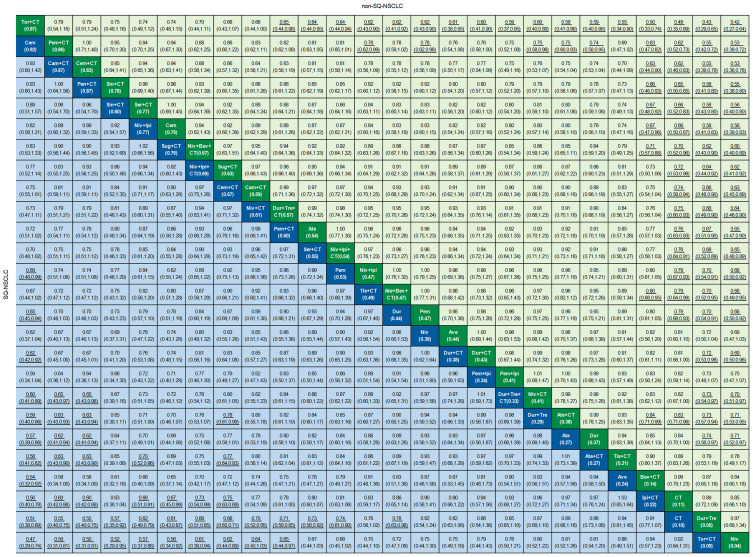
Pooled OS estimates of multiple comparisons in network meta-analysis. Treatment regimens are presented in order of OS ranking from high to low according to SUCRAs. Dark blue represents the regimens for SQ-NSCLC (SUCRA values), and dark green represents the regimens for non-SQ-NSCLC (SUCRA values). Data are HRs (95% CrIs) for column-defined treatment versus row-defined treatment for SQ-NSCLC (lower triangle) and row-defined treatment versus column-defined treatment for non-SQ-NSCLC (upper triangle). Significant results are underlined.OS, overall survival; SUCRA, surface under the cumulative ranking; HR, hazard ratio; CI, confidence interval; SQ-NSCLC, squamous non-small cell lung cancer; Anl, anlotinib; Ate, atezolizumab; Ave, avelumab; Ben, benmelstobart; Bev, bevacizumab; Cam, camrelizumab; Cem, cemiplimab; Dur, durvalumab; Ipi, ipilimumab; Ivo, ivonescimab; Niv, nivolumab; Pem, pembrolizumab; Pen, penpulimab; Ser, serplulimab; Sin, sintilimab; Sug, sugemalimab; Tis, tislelizumab; Tor, toripalimab; Tre, tremelimumab; CT, chemotherapy.

**Figure 4:**
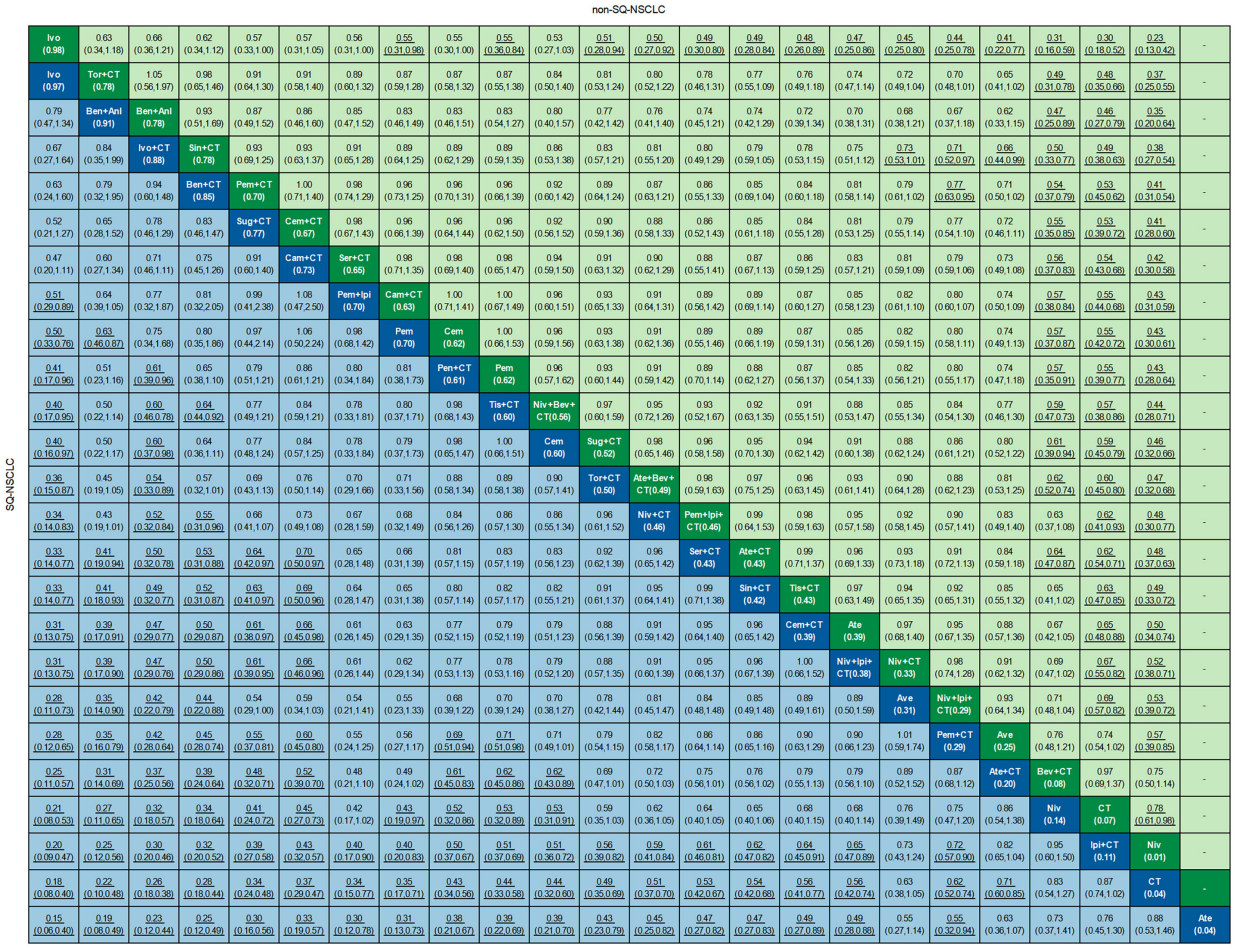
Pooled PFS estimates of multiple comparisons in network meta-analysis. Treatment regimens are presented in order of PFS ranking from high to low according to SUCRAs. Dark blue represents the regimens for SQ-NSCLC (SUCRA values), and dark green represents the regimens for non-SQ-NSCLC (SUCRA values). Data are HRs (95% CrIs) for column-defined treatment versus row-defined treatment for SQ-NSCLC (lower triangle) and row-defined treatment versus column-defined treatment for non-SQ-NSCLC (upper triangle). Significant results are underlined.PFS, progression-free survival; SUCRA, surface under the cumulative ranking; HR, hazard ratio; CI, confidence interval; SQ-NSCLC, squamous non-small cell lung cancer; Anl, anlotinib; Ate, atezolizumab; Ave, avelumab; Ben, benmelstobart; Bev, bevacizumab; Cam, camrelizumab; Cem, cemiplimab; Dur, durvalumab; Ipi, ipilimumab; Ivo, ivonescimab; Niv, nivolumab; Pem, pembrolizumab; Pen, penpulimab; Ser, serplulimab; Sin, sintilimab; Sug, sugemalimab; Tis, tislelizumab; Tor, toripalimab; Tre, tremelimumab; CT, chemotherapy.

**Figure 5:**
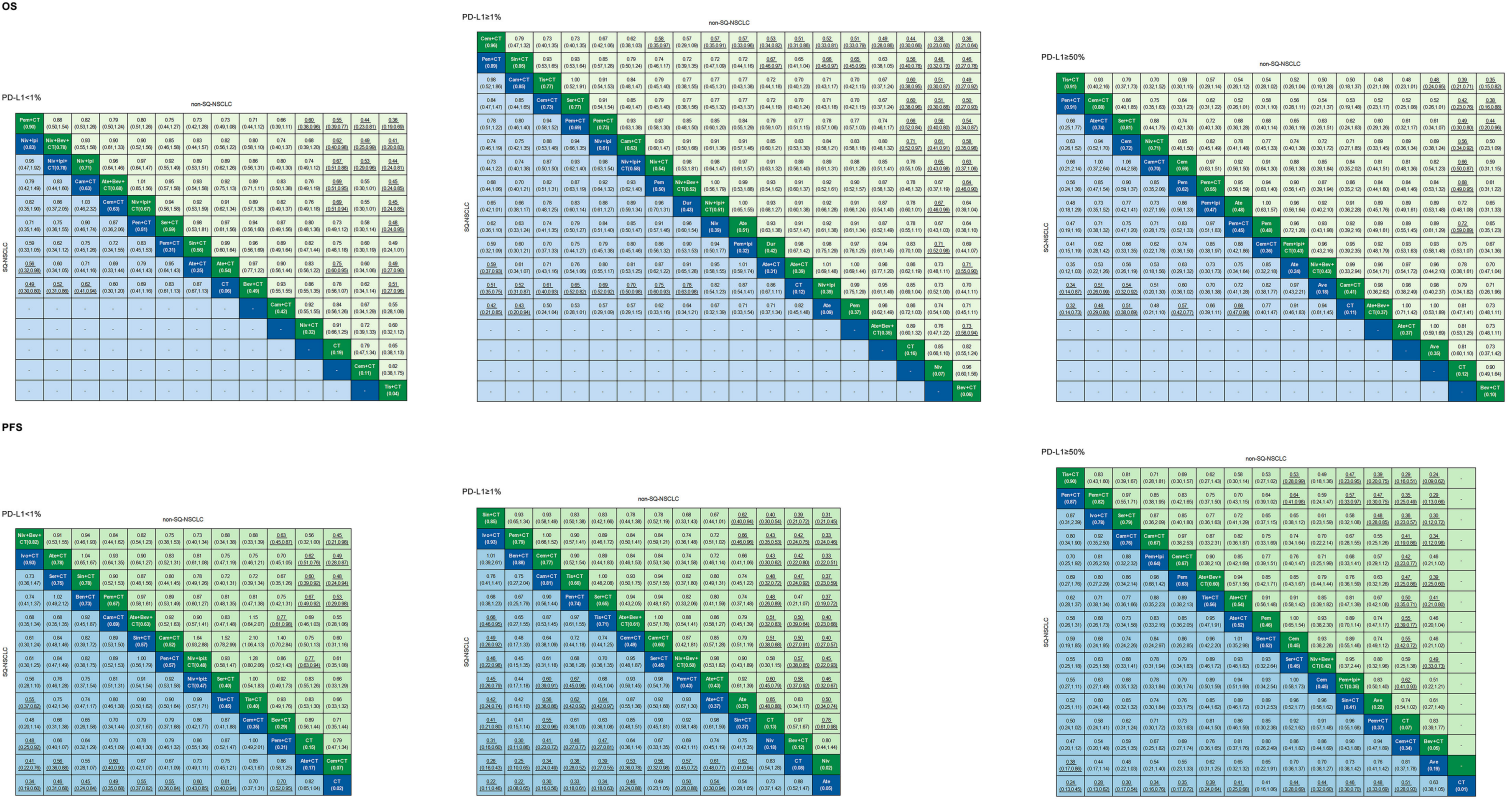
Subgroup network meta-analysis of OS and PFS based on PD-L1 expression. Treatment regimens are presented in order of OS or PFS ranking from high to low according to SUCRAs. Dark blue represents the regimens for SQ-NSCLC (SUCRA values), and dark green represents the regimens for non-SQ-NSCLC (SUCRA values). Data are HRs (95% CrIs) for column-defined treatment versus row-defined treatment for SQ-NSCLC (lower triangle) and row-defined treatment versus column-defined treatment for non-SQ-NSCLC (upper triangle). Significant results are underlined.OS, overall survival; PFS, progression-free survival; SUCRA, surface under the cumulative ranking; HR, hazard ratio; CI, confidence interval; SQ-NSCLC, squamous non-small cell lung cancer; Anl, anlotinib; Ate, atezolizumab; Ave, avelumab; Ben, benmelstobart; Bev, bevacizumab; Cam, camrelizumab; Cem, cemiplimab; Dur, durvalumab; Ipi, ipilimumab; Ivo, ivonescimab; Niv, nivolumab; Pem, pembrolizumab; Pen, penpulimab; Ser, serplulimab; Sin, sintilimab; Sug, sugemalimab; Tis, tislelizumab; Tor, toripalimab; Tre, tremelimumab; CT, chemotherapy.

The networks of eligible comparisons are shown in **Figure 2**. Results of the network meta-analysis are presented in **Figures 3** (OS) and **4** (PFS). Subgroup analysis results are shown in **Figure 5** the trials and treatment arms included in each subgroup are presented in **Supplementary Table 2**. Results for grade 3–5 TRAEs are presented in **Supplementary Figure 1**. The SUCRA values for each regimen are presented in **Supplementary Figures 2** (OS) and **3** (PFS), and also shown in parentheses next to the regimen in **Figures 3**–**6** and **Supplementary Figure 1**; the ranking curves are displayed in **Supplementary Figures 4**–**S8**.

#### 3.2.1 Overall survival

For non-SQ-NSCLC (30 trials involving 13, 719 patients and 24 ICI-based treatments), toripalimab plus chemotherapy was more effective than most other ICI regimens (including atezolizumab, atezolizumab plus chemotherapy ± bevacizumab, avelumab, durvalumab, durvalumab plus chemotherapy or tremelimumab, nivolumab, nivolumab plus chemotherapy or ipilimumab, pembrolizumab ± ipilimumab, and tislelizumab plus chemotherapy), with HRs ranging from 0.42 to 0.65; it was ranked as the best treatment (SUCRA = 0.97). In contrast, for SQ-NSCLC (28 trials involving 8, 129 patients and 24 ICI-based treatments), toripalimab plus chemotherapy was inferior to many other ICI regimens (including cemiplimab ± chemotherapy, camrelizumab plus chemotherapy, nivolumab plus chemotherapy or ipilimumab, pembrolizumab plus chemotherapy, penpulimab plus chemotherapy, and sintilimab plus chemotherapy), with HRs for comparators versus toripalimab plus chemotherapy ranging from 0.47 to 0.65; it was ranked as the least effective treatment (SUCRA = 0.09), while cemiplimab was ranked as the best regimen (SUCRA = 0.92). In addition, pembrolizumab plus chemotherapy was superior to seven other ICI regimens for non-SQ-NSCLC (including atezolizumab plus chemotherapy, durvalumab ± tremelimumab, nivolumab, nivolumab plus chemotherapy or ipilimumab, and pembrolizumab), with HRs ranging from 0.53 to 0.78, and was ranked as the second-best treatment (SUCRA = 0.86); however, this regimen was only better than toripalimab plus chemotherapy in SQ-NSCLC (HR = 0.65, 95% CrI: 0.44–0.97) and had only a moderate efficacy ranking (SUCRA = 0.60).

#### 3.2.2 Progression-free survival

For both SQ-NSCLC (24 trials involving 7, 463 patients and 23 ICI-based treatments; SUCRA = 0.97) and non-SQ-NSCLC (24 trials involving 9, 454 patients and 21 ICI-based treatments; SUCRA = 0.98), ivonescimab achieved significantly longer PFS than most other ICI regimens and was ranked as the best treatment. Benmelstobart plus anlotinib was also more effective than many comparators. Toripalimab plus chemotherapy was ranked as the second-best regimen for non-SQ-NSCLC (SUCRA = 0.78), but achieved only moderate ranking for SQ-NSCLC (SUCRA = 0.50).

#### 3.2.3 Subgroup analysis for overall survival by PD-L1 expression

##### 3.2.3.1 PD-L1 <1%

For SQ-NSCLC (data from 7 trials involving 1, 047 patients and 7 ICI combination regimens), nivolumab plus ipilimumab was associated with prolonged OS compared with atezolizumab plus chemotherapy (HR = 0.56, 95% CrI: 0.32–0.98) and showed a trend toward improved OS compared with pembrolizumab plus chemotherapy (HR = 0.59, 95% CrI: 0.33–1.05); it was ranked as the most effective regimen (SUCRA = 0.83). Pembrolizumab plus chemotherapy had a low efficacy ranking (SUCRA = 0.31). For non-SQ-NSCLC (data from 14 trials involving 2, 908 patients and 12 ICI combination regimens), pembrolizumab plus chemotherapy was superior to nivolumab plus chemotherapy, tislelizumab plus chemotherapy, and cemiplimab plus chemotherapy (HR range: 0.36–0.60) and was ranked as the best treatment (SUCRA = 0.90). Although nivolumab plus ipilimumab was also better than tislelizumab plus chemotherapy and cemiplimab plus chemotherapy (HR range: 0.44–0.67), it was ranked as the third-best treatment (SUCRA = 0.71).

##### 3.2.3.2 PD-L1 ≥ 1% (excluding trials restricted to PD-L1 ≥ 50%)

In SQ-NSCLC (data from 13 trials involving 2, 867 patients and 12 ICI-based treatments), pembrolizumab and durvalumab monotherapies were not inferior to any combination regimen. In non-SQ-NSCLC (data from 19 trials involving 6, 105 patients and 16 ICI-based treatments), cemiplimab plus chemotherapy and sintilimab plus chemotherapy were associated with improved OS compared with pembrolizumab, durvalumab, and nivolumab monotherapies (HR range: 0.38–0.67). Penpulimab plus chemotherapy ranked highest for SQ-NSCLC (SUCRA = 0.89), while cemiplimab plus chemotherapy was the top-ranked regimen for non-SQ-NSCLC (SUCRA = 0.96).

##### 3.2.3.3 PD-L1 ≥ 50%

For both SQ-NSCLC (data from 11 trials involving 1, 290 patients and 10 ICI-based treatments) and non-SQ-NSCLC (data from 17 trials involving 2, 818 patients and 14 ICI-based treatments), all ICI monotherapies (except avelumab) were not inferior to any combination regimen. Penpulimab plus chemotherapy (SUCRA = 0.91) and atezolizumab plus chemotherapy (SUCRA = 0.74) were the top two regimens for SQ-NSCLC, with cemiplimab plus chemotherapy being the least effective combination (SUCRA = 0.36). Conversely, in non-SQ-NSCLC, tislelizumab plus chemotherapy (SUCRA = 0.91) and cemiplimab plus chemotherapy (SUCRA = 0.88) ranked highest, whereas atezolizumab plus chemotherapy was the least effective combination (SUCRA = 0.37).

#### 3.2.4 Subgroup analysis for progression-free survival by PD-L1 expression

##### 3.2.4.1 PD-L1 < 1%

In SQ-NSCLC (data from 11 trials involving 1, 931 patients and 11 ICI combination regimens), ivonescimab plus chemotherapy was more effective than pembrolizumab plus chemotherapy, atezolizumab plus chemotherapy, and tislelizumab plus chemotherapy (HR range: 0.41–0.55) and ranked highest (SUCRA = 0.93); cemiplimab plus chemotherapy showed no significant difference compared with any other regimen. In non-SQ-NSCLC (data from 12 trials involving 2, 507 patients and 10 ICI combination regimens), cemiplimab plus chemotherapy was inferior to four other ICI treatments (including nivolumab plus bevacizumab plus chemotherapy, atezolizumab plus chemotherapy, pembrolizumab plus chemotherapy, and sintilimab plus chemotherapy), with HRs for comparators versus cemiplimab plus chemotherapy ranging from 0.45 to 0.53; it was ranked as the least effective treatment (SUCRA = 0.07), while nivolumab plus bevacizumab plus chemotherapy was the top-ranked regimen (SUCRA = 0.82).

##### 3.2.4.2 PD-L1 ≥ 1% (excluding trials restricted to PD-L1 ≥ 50%)

In SQ-NSCLC (data from 12 trials involving 2, 222 patients and 12 ICI-based treatments), ivonescimab plus chemotherapy outperformed six other ICI combinations (including tislelizumab plus chemotherapy, cemiplimab plus chemotherapy, serplulimab plus chemotherapy, pembrolizumab plus chemotherapy, atezolizumab plus chemotherapy, and sintilimab plus chemotherapy), with HRs ranging from 0.41 to 0.66; it was the top-ranked regimen (SUCRA = 0.93). Sintilimab plus chemotherapy ranked as the least effective combination (SUCRA = 0.37). In non-SQ-NSCLC (data from 13 trials involving 3, 414 patients and 11 ICI-based treatments), no significant differences were observed among ICI combination treatments, and sintilimab plus chemotherapy ranked highest (SUCRA = 0.85).

##### 3.2.4.3 PD-L1 ≥ 50%

In SQ-NSCLC (data from 14 trials involving 1, 632 patients and 14 ICI-based treatments), pembrolizumab and cemiplimab monotherapies were non-inferior to any combination regimen; penpulimab plus chemotherapy was the top-ranked regimen (SUCRA = 0.87), while tislelizumab plus chemotherapy (SUCRA = 0.56) and pembrolizumab plus chemotherapy (SUCRA = 0.37) had moderate or low efficacy rankings. In non-SQ-NSCLC (data from 14 trials involving 1, 994 patients and 12 ICI-based treatments), tislelizumab plus chemotherapy and pembrolizumab plus chemotherapy outperformed cemiplimab monotherapy (HR range: 0.53–0.64); the two combination regimens had the highest rankings (SUCRA = 0.90 and 0.82, respectively).

#### 3.2.5 Grade 3–5 treatment-related adverse events

A total of 38 trials comprising 23, 065 patients were included in the safety analysis. Nivolumab, atezolizumab, durvalumab, pembrolizumab, and cemiplimab monotherapies were associated with significantly lower incidences of grade 3–5 TRAEs compared with all ICI combination therapies (OR range: 0.10–0.61). Ivonescimab and avelumab monotherapies were more toxic than other ICI monotherapies but less toxic than many combination regimens.

Among ICI combinations, durvalumab plus tremelimumab demonstrated a lower risk of grade 3–5 TRAEs than all other regimens (OR range: 0.25–0.62) except toripalimab plus chemotherapy. Toripalimab plus chemotherapy, nivolumab plus ipilimumab, and durvalumab plus chemotherapy were also less toxic than many comparators.

Based on SUCRA rankings, nivolumab (SUCRA = 0.99), atezolizumab (SUCRA = 0.95), and durvalumab (SUCRA = 0.94) were the three least toxic regimens overall. Among combination treatments, durvalumab plus tremelimumab (SUCRA = 0.82), toripalimab plus chemotherapy (SUCRA = 0.70), and nivolumab plus ipilimumab (SUCRA = 0.68) were the least toxic. Conversely, ipilimumab plus chemotherapy (SUCRA = 0.11), cemiplimab plus chemotherapy (SUCRA = 0.13), and benmelstobart plus chemotherapy (SUCRA = 0.14) were the three most toxic regimens.

### 3.3 Inconsistency assessment and sensitivity analysis

The consistency model demonstrated a similar fit to the inconsistency model (**Supplementary Table 3**), indicating no significant global inconsistency. Local consistency was supported by consistent results between pairwise and network meta-analyses (**Supplementary Table 4**). Only one independent closed loop was present in the network, and node-splitting analysis showed no significant inconsistency between direct and indirect evidence (**Supplementary Table 5**). Most direct comparisons were informed by a single trial, precluding formal assessment of between-study heterogeneity for those comparisons. For the few comparisons informed by two or more trials, heterogeneity was generally low (I² range: 0%–44%), with the exception of pembrolizumab vs. chemotherapy for OS in non-SQ-NSCLC, which showed substantial heterogeneity (I² = 74%) (**Supplementary Table 6**).

Sensitivity analysis performed using a random-effects model (**Supplementary Figures 9**, **10**) or restricting the analysis to trials conducted exclusively in SQ-NSCLC or non-SQ-NSCLC (i.e., omitting mixed-histology trials with subgroup data) (**Supplementary Figure 11**) revealed no substantial changes in treatment ranking order for OS or PFS compared with the main analysis.

### 3.4 Risk of bias assessment

Nineteen trials (9, 13, 14, 24, 25, 35–45, 47, 48, 51–53) and one trial (50) were assessed as having low and unclear risk of bias, respectively. The remaining 23 trials were classified as having a high risk of bias, primarily due to their open-label design resulting in lack of blinding of participants and personnel (**Supplementary Figure 12**).

## 4 Discussion

In this study, we investigated the comparative efficacy of currently available first-line ICI-based regimens separately in patients with advanced SQ-NSCLC and non-SQ-NSCLC. Our findings suggest the presence of histology-specific differences in the survival benefits of individual ICI regimens. For example, among patients with non-SQ-NSCLC, toripalimab plus chemotherapy and pembrolizumab plus chemotherapy achieved significantly longer OS than many other ICI regimens and were ranked as the two best treatments. In contrast, for OS in SQ-NSCLC, toripalimab plus chemotherapy was inferior to many other ICI regimens and was ranked as the least effective treatment, whereas pembrolizumab plus chemotherapy exhibited only moderate efficacy.

These histology-specific differences in OS benefit appeared to persist across subgroup analyses stratified by PD-L1 expression level. In the PD-L1 < 1% subgroup, nivolumab plus ipilimumab showed a trend toward improved OS compared with pembrolizumab plus chemotherapy and was ranked as the best regimen for SQ-NSCLC; for non-SQ-NSCLC, however, pembrolizumab plus chemotherapy ranked as the most effective, whereas nivolumab plus ipilimumab yielded only moderate efficacy. Although the precise mechanisms underlying these observations remain to be fully elucidated, differences in the clinical and biological characteristics between SQ-NSCLC and non-SQ-NSCLC likely play an important role. Compared with non-SQ-NSCLC, squamous histology is associated with lower infiltration of tumor-infiltrating lymphocytes (TILs) but higher infiltration of neutrophils, which are known to suppress T-cell activity (66). The addition of anti-CTLA-4 therapy may lower the threshold for T-cell activation and enhance antitumor efficacy (67, 68). Moreover, patients with SQ-NSCLC often have a history of smoking and a high tumor mutational burden (TMB), which has been associated with greater benefit from dual ICI therapy (5, 29). In the CheckMate 227 trial (5), patients with PD-L1 < 1% and TMB ≥ 10 mut/Mb derived greater OS benefit from nivolumab plus ipilimumab compared with those with TMB < 10 mut/Mb (HR = 0.51, 95% CI: 0.30–0.87 vs. HR = 0.69, 95% CI: 0.46–1.05). Similarly, in the NEPTUNE trial (29), although OS was comparable between durvalumab plus tremelimumab and chemotherapy in the overall PD-L1 < 1% population, subgroup analysis suggested improved OS with dual ICI in patients with PD-L1 < 1% and TMB ≥ 20 mut/Mb.

For non-SQ-NSCLC with PD-L1 < 1%, ICI combined with chemotherapy may be more effective, as chemotherapy-induced tumor cell death can release antigens and potentiate the immune response. However, it is noteworthy that although pembrolizumab plus chemotherapy demonstrated relatively favorable efficacy compared with nivolumab plus ipilimumab in this population, other ICI-plus-chemotherapy regimens, such as cemiplimab plus chemotherapy and tislelizumab plus chemotherapy, yielded inferior OS relative to nivolumab plus ipilimumab. This observation suggests that the choice of ICI agent and its synergy with chemotherapy critically influence survival outcomes, underscoring the potential need for biomarker-guided regimen selection. For example, non-SQ-NSCLC is associated with a higher frequency of *KEAP1* and/or *STK11* mutations compared with squamous histology (28); patients harboring these alterations may derive greater benefit from dual ICI therapy than from ICI plus chemotherapy (69). Thus, dual ICI rather than ICI plus chemotherapy could be a preferable strategy in patients with non-SQ-NSCLC harboring *KEAP1* and/or *STK11* mutations.

In the PD-L1 ≥ 1% and ≥ 50% subgroups, although ICI plus chemotherapy generally outperformed ICI monotherapy in both histologies, the comparative efficacy of individual combination regimens varied by histological subtype. For example, in the PD-L1 ≥ 50% subgroup, atezolizumab plus chemotherapy ranked as the second-best treatment for SQ-NSCLC but was the least effective combination for non-SQ-NSCLC; conversely, cemiplimab plus chemotherapy was the least effective combination for SQ-NSCLC but ranked second for non-SQ-NSCLC. In the PD-L1 ≥ 1% subgroup, cemiplimab plus chemotherapy showed moderate efficacy in SQ-NSCLC but ranked as the best regimen in non-SQ-NSCLC. Together, these findings suggest that PD-L1 expression alone may be insufficient to guide treatment selection and that histological subtype should be integrated into clinical decision-making to optimize therapeutic outcomes.

The IMpower150 (19) and TASUKI-52 (9) trials have demonstrated enhanced ICI efficacy with the addition of bevacizumab, a vascular endothelial growth factor (VEGF) monoclonal antibody, in patients with non-SQ-NSCLC. Consistently, our meta-analysis showed superior efficacy of nivolumab plus bevacizumab and chemotherapy for non-SQ-NSCLC, particularly in the low PD-L1 subgroup. However, bevacizumab is not recommended for SQ-NSCLC due to the high risk of life-threatening treatment-related adverse events (TRAEs), including fatal bleeding (70). Nevertheless, several recent phase 3 trials have reported promising efficacy for ICIs combined with VEGF-targeted agents in the first-line treatment of advanced SQ-NSCLC. In the CAMPASS trial (51), benmelstobart (an anti-PD-L1 antibody) plus anlotinib (an anti-angiogenic multi-kinase inhibitor) significantly improved PFS compared with pembrolizumab in advanced PD-L1-positive NSCLC, with a more pronounced benefit in SQ-NSCLC (HR = 0.63, 95% CI: 0.46–0.86) than in non-SQ-NSCLC (HR = 0.83, 95% CI: 0.54–1.27). In the HARMONi-2 trial (52), ivonescimab (a bispecific antibody targeting both PD-1 and VEGF) demonstrated prolonged PFS compared with pembrolizumab in PD-L1-positive NSCLC across histological subtypes. In the HARMONi-6 trial (53), which enrolled exclusively patients with SQ-NSCLC, ivonescimab plus chemotherapy achieved superior PFS to tislelizumab plus chemotherapy, regardless of PD-L1 expression. In our meta-analysis, ivonescimab and benmelstobart plus anlotinib ranked as the two best treatments for PFS in both SQ-NSCLC and non-SQ-NSCLC. In subgroup analyses, ivonescimab plus chemotherapy provided the greatest PFS benefit in the PD-L1 < 1% and ≥ 1% subgroups for SQ-NSCLC. Nonetheless, these trials were conducted exclusively in China, had relatively short follow-up periods, and lacked mature OS data. Therefore, the efficacy of these novel agents requires confirmation in large, global RCTs with long-term survival outcomes.

Regarding safety, ICI monotherapies were associated with a significantly lower risk of grade 3–5 TRAEs compared with ICI combination regimens. Among combination treatments, dual ICI therapy was generally less toxic than ICI plus chemotherapy. However, certain ICI-plus-chemotherapy regimens, such as toripalimab plus chemotherapy and durvalumab plus chemotherapy, exhibited toxicity profiles comparable to dual ICI and were less toxic than many other ICI-plus-chemotherapy combinations. These safety findings may assist clinicians in selecting individualized treatment strategies based on benefit–risk assessment. For example, ICI monotherapies (e.g., cemiplimab, pembrolizumab, and ivonescimab) could represent suitable alternatives for patients with high PD-L1 expression and poor performance status, given their favorable safety profile and non-inferior efficacy compared with combination regimens.

Several previous network meta-analyses have examined the comparative efficacy of individual ICI regimens in advanced SQ-NSCLC or non-SQ-NSCLC (71–77). As summarized in **Supplementary Table 7**, most of these studies included a limited number of trials and treatment regimens and did not incorporate recently updated long-term survival data. In addition, none of these studies evaluated novel agents such as ivonescimab and benmelstobart. Furthermore, all previous meta-analyses assessed efficacy either in SQ-NSCLC or in non-SQ-NSCLC, but no study simultaneously examined both histological subtypes. Consequently, none of them could assess whether the relative efficacy of a given ICI regimen differs between SQ-NSCLC and non-SQ-NSCLC.

Our study has several strengths. First, we assessed the comparative efficacy of first-line ICI regimens separately in SQ-NSCLC and non-SQ-NSCLC, focusing on histology-specific differences in the efficacy of individual ICI regimens. Second, we incorporated the latest evidence, including newly published trials and updated long-term survival data, and evaluated the efficacy of two novel ICI-based treatments (ivonescimab and benmelstobart). Third, we conducted subgroup analyses based on PD-L1 expression level. These features enhance the stability and credibility of our results and may help guide clinicians in developing individualized ICI treatment strategies for patients with advanced SQ-NSCLC and non-SQ-NSCLC.

This study also has several limitations. First, most ICI-based regimens were compared indirectly, and the majority of direct treatment comparisons were based on evidence from a single trial; as a result, heterogeneity for these comparisons could not be formally assessed. In addition, many trials enrolled unselected NSCLC populations, and data for SQ-NSCLC and non-SQ-NSCLC were derived from subgroup analyses. Transitivity could not be formally assessed because most trials did not report patient clinical characteristics separately by histology. Accordingly, the results should be interpreted with caution due to potential confounding. Nevertheless, all the studies were phase 3 RCTs with predefined subgroup analyses by histology; key patient characteristics, such as ECOG performance status 0–1, absence of sensitizing *EGFR*/*ALK* alterations, and absence of symptomatic brain metastases, were generally consistent across trials. In addition, a sensitivity analysis restricted to trials conducted exclusively in SQ-NSCLC or non-SQ-NSCLC yielded treatment rankings similar to those of the main analysis. Subgroup analyses by PD-L1 expression further supported the presence of histology-specific efficacy differences across PD-L1 levels. Second, due to insufficient data or lack of connected treatment networks, we were unable to evaluate the comparative efficacy of certain ICI regimens in all PD-L1 subgroups. For example, trials limited to PD-L1 ≥ 1% or ≥ 50% populations did not provide data for the PD-L1 < 1% subgroup. Although the HARMONi-2 trial reported outcomes for PD-L1 ≥ 50%, ivonescimab could not be positioned within the network due to the absence of direct or indirect comparators. Third, other potential effect modifiers, such as age, sex, smoking status, region, and PD-L1 assay methodology, could not be examined in subgroup analyses due to data limitations.

In conclusion, the efficacy of individual first-line ICI regimens in advanced NSCLC may differ substantially by histological subtype, and these differences were observed across PD-L1 expression levels in our analysis. Our findings suggest that PD-L1 status alone may be insufficient to guide treatment selection and that histological subtype should be incorporated into clinical decision-making to optimize therapeutic outcomes. Future trial designs could consider both PD-L1 expression and pathological type to refine treatment strategies and enable more personalized approaches.

## Data Availability

The original contributions presented in the study are included in the article/**Supplementary Material**. Further inquiries can be directed to the corresponding authors.
