# Supplementary material for: Effect of histology on the efficacy of first-line immune checkpoint inhibitors in advanced non-small cell lung cancer: a systematic review and network meta-analysis
Source: Front Immunol. 2026 Jun 3;17:1850384. doi: 10.3389/fimmu.2026.1850384 (PMC13272326; doi:10.3389/fimmu.2026.1850384)
Supplement: Supplementary file 1 [file DataSheet1.docx]

**Table S1** Search strategy.

**a:** Search strategy in PubMed

| # | Query |
| --- | --- |
| #1 | "Carcinoma, Non-Small-Cell Lung"[mh] |
| #2 | "nsclc"[Title/Abstract] |
| #3 | "lung cancer*"[Title/Abstract] OR "lung carcinoma*"[Title/Abstract] OR "lung neoplasm*"[Title/Abstract] OR "lung tumor*"[Title/Abstract] OR "lung tumour*"[Title/Abstract] |
| #4 | "non small cell*"[Title/Abstract] OR "nonsmall cell*"[Title/Abstract] |
| #5 | #3 AND #4 |
| #6 | #1 OR #2 OR #5 |
| #7 | "Immunotherapy"[Mesh] |
| #8 | "Immune Checkpoint Inhibitors"[Mesh] |
| #9 | "Immunotherapies"[Title/Abstract] OR "programmed death ligand 1"[Title/Abstract] OR "PD-L1"[Title/Abstract] OR "programmed death 1"[Title/Abstract] OR "PD-1"[Title/Abstract] OR "anti programmed death ligand 1"[Title/Abstract] OR "Anti-PD-L1"[Title/Abstract] OR "anti programmed death 1"[Title/Abstract] OR "Anti-PD-1"[Title/Abstract] OR "Anti-CTLA-4"[Title/Abstract] OR "Atezolizumab"[Title/Abstract] OR "Durvalumab"[Title/Abstract] OR "Nivolumab"[Title/Abstract] OR "Pembrolizumab"[Title/Abstract] OR "Camrelizumab"[Title/Abstract] OR "Sintilimab"[Title/Abstract] OR "Cemiplimab"[Title/Abstract] OR "Tislelizumab"[Title/Abstract] OR "Ipilimumab"[Title/Abstract] OR "Avelumab"[Title/Abstract] OR "Tremelimumab"[Title/Abstract] OR "Sugemalimab"[Title/Abstract] OR "Serplulimab"[Title/Abstract] OR "Toripalimab"[Title/Abstract] |
| #10 | #7 OR #8 OR #9 |
| #11 | "Advanced"[Title/Abstract] OR "stage 4"[Title/Abstract] OR "stage iv"[Title/Abstract] OR "metasta*"[Title/Abstract] |
| #12 | ("randomized controlled trial"[Publication Type]) OR (((RCT[Title/Abstract]) OR ("randomized controlled trial"[Title/Abstract]))) |
| #13 | #6 AND #10 AND #11 AND #12 |

**b:** Search strategy in Embase

| # | Query |
| --- | --- |
| #1 | ‘non small cell lung cancer’/exp |
| #2 | ’nsclc’:ab,ti |
| #3 | 'non small cell*':ab,ti OR 'nonsmall cell*':ab,ti |
| #4 | 'lung cancer*':ab,ti OR 'lung carcinoma*':ab,ti OR 'lung neoplasm*':ab,ti OR 'lung tumor*':ab,ti OR 'lung tumour*':ab,ti |
| #5 | #3 AND #4 |
| #6 | #1 OR #2 OR #5 |
| #7 | ‘immunotherapy'/exp |
| #8 | 'immune checkpoint inhibitor'/exp |
| #9 | ‘programmed death ligand 1’:ab,ti OR ‘PD-L1’:ab,ti OR ‘programmed death 1’:ab,ti OR ‘PD-1’:ab,ti OR ‘anti-programmed death ligand 1’:ab,ti OR ‘anti–PD-L1’:ab,ti OR ‘anti-programmed death 1’:ab,ti OR ‘anti–PD-1’:ab,ti OR ‘atezolizumab’:ab,ti OR ‘durvalumab’:ab,ti OR ‘nivolumab’:ab,ti OR ‘pembrolizumab’:ab,ti OR ‘camrelizumab’:ab,ti OR ‘sintilimab’:ab,ti OR ‘cemiplimab’:ab,ti OR ‘avelumab’:ab,ti OR ‘tislelizumab’:ab,ti OR ‘anti–CTLA-4’:ab,ti OR ‘ipilimumab’:ab,ti OR ‘tremelimumab’:ab,ti OR ‘sugemalimab’:ab,ti OR ‘serplulimab’:ab,ti OR ‘toripalimab’:ab,ti |
| #10 | #7 OR #8 OR #9 |
| #11 | 'advanced':ab,ti OR 'stage 4':ab,ti OR 'stage iv':ab,ti OR 'metasta*':ab,ti |
| #12 | 'controlled clinical trial'/de OR 'randomized controlled trial'/de |
| #13 | #6 AND #10 AND #11 AND #12 |

**c:** Search strategy in Cochrane Library

| # | Query |
| --- | --- |
| #1 | MeSH descriptor: [Carcinoma, Non-Small-Cell Lung] explode all trees |
| #2 | nsclc |
| #3 | lung cancer* |
| #4 | lung carcinom* |
| #5 | lung neoplasm* |
| #6 | lung tumor* |
| #7 | lung tumour* |
| #8 | non small cell* |
| #9 | nonsmall cell* |
| #10 | #3 OR #4 OR #5 OR #6 OR #7 |
| #11 | #8 OR #9 |
| #12 | #10 AND #11 |
| #13 | #1 OR #2 OR #12 |
| #14 | MeSH descriptor: [Immunotherapy] explode all trees |
| #15 | MeSH descriptor: [Immune Checkpoint Inhibitors] explode all trees |
| #16 | (programmed death ligand 1 OR PD-L1 OR programmed death 1 OR PD-1 OR anti-programmed death ligand 1 OR anti–PD-L1 OR anti-programmed death 1 OR anti–PD-1 OR atezolizumab OR durvalumab OR nivolumab OR pembrolizumab OR camrelizumab OR sintilimab OR cemiplimab OR avelumab OR tislelizumab OR anti–CTLA-4 OR ipilimumab OR tremelimumab OR sugemalimab OR serplulimab OR toripalimab):ti,ab,kw |
| #17 | #14 OR #15 OR #16 |
| #18 | (advanced OR "stage 4" OR "stage IV" OR metasta*):ti,ab,kw |
| #19 | #13 AND #17 AND #18 |

**d:** Search strategy in Web of Science

| # | Query |
| --- | --- |
| #1 | TS=("lung cancer" OR "non-small cell lung cancer" OR NSCLC OR ((lung OR pulmon*) AND (neoplas* OR cancer OR carcinoma* OR tumour* or tumor*) AND ((“non small cell*”) OR (“nonsmall cell*”)))) |
| #2 | TS=(“programmed death ligand 1” OR “PD-L1” OR “programmed death 1” OR “PD-1” OR “anti-programmed death ligand 1” OR “anti–PD-L1” OR “anti-programmed death 1” OR “anti–PD-1” OR “atezolizumab” OR “durvalumab” OR “nivolumab” OR “pembrolizumab” OR “camrelizumab” OR “sintilimab” OR “tislelizumab” OR “cemiplimab” OR “avelumab” OR “tislelizumab” OR “anti–CTLA-4” OR “ipilimumab” OR “tremelimumab” OR “sugemalimab” OR “serplulimab” OR “toripalimab”) |
| #3 | TS=("advanced" OR "stage 4" OR "stage iv" OR "metasta*") |
| #4 | TS=("randomized controlled trial" OR "controlled clinical trial") |
| #5 | #1 AND #2 AND #3 AND #4 |

**e:** Search strategy in Scopus

| # | Query |
| --- | --- |
| #1 | TITLE-ABS-KEY ({Carcinoma, Non-Small-Cell Lung}) |
| #2 | TITLE-ABS-KEY (nsclc) |
| #3 | #1 OR #2 |
| #4 | TITLE-ABS-KEY ({Immunotherapy} OR {Immune Checkpoint Inhibitors}) |
| #5 | TITLE-ABS ("Immunotherapies" OR "programmed death ligand 1" OR "PD-L1" OR "programmed death 1" OR "PD-1" OR "anti programmed death ligand 1" OR "Anti-PD-L1" OR "anti programmed death 1" OR "Anti-PD-1" OR "Anti-CTLA-4" OR "Atezolizumab" OR "Durvalumab" OR "Nivolumab" OR "Pembrolizumab" OR "Camrelizumab" OR "Sintilimab" OR "Cemiplimab" OR "Tislelizumab" OR "Ipilimumab" OR "Avelumab" OR "Tremelimumab" OR "Sugemalimab" OR "Serplulimab" OR "Toripalimab") |
| #6 | #4 OR #5 |
| #7 | TITLE-ABS ("Advanced" OR "stage 4" OR "stage iv" OR "metasta*") |
| #8 | TITLE-ABS-KEY ("randomized controlled trial") |
| #9 | TITLE-ABS-KEY ("controlled clinical trial") |
| #10 | #8 OR #9 |
| #11 | #3 AND #6 AND #7 AND #10 |

**Table S2** Treatment and outcome of trials included in each PD-L1 subgroup

|  | **Subgroup of PD-L1<1%** | | |  | **Subgroup of PD-L1≥1%** | | |  | **Subgroup of PD-L1≥50%** | | |
| --- | --- | --- | --- | --- | --- | --- | --- | --- | --- | --- | --- |
|  | **Trial included** | **Treatment (sample size)** | **HR (95%CI)** |  | **Trial included** | **Treatment (sample size)** | **HR (95%CI)** |  | **Trial included** | **Treatment (sample size)** | **HR (95%CI)** |
| **OS (SQ- NSCLC)** | CheckMate- 227/2019[5] | Nivolumab+ipilimumab/CT (46/46) | 0.49 (0.30-0.79) |  | CheckMate- 026/2017[4] | Nivolumab/CT (65/64) | 0.82 (0.54-1.24) |  | Keynote-024/ 2019[11] | Pembrolizumab/CT  (29/27) | 0.73 (0.38-1.39) |
|  | CheckMate- 9LA/2025[8] | Nivolumab+ipilimumab+CT/CT (36/36) | 0.52 (0.31-0.86) |  | CheckMate- 227/2019[5] | Nivolumab+ipilimumab/CT(117/116) | 0.69 (0.52-0.92) |  | Keynote-042/ 2019[12] | Pembrolizumab/CT (107/114) | 0.53  (0.38-0.75) |
|  | Keynote-407/2023[14] | Pembrolizumab+CT/CT(95/99) | 0.83 (0.61-1.13) |  | CheckMate- 9LA/2025[8] | Nivolumab+ipilimumab+CT/CT (74/74) | 0.70 (0.50-0.99) |  | Keynote-407/ 2023[14] | Pembrolizumab+CT/CT  (73/73) | 0.68  (0.47-0.97) |
|  | IMpower-131 /2020[17] | Atezolizumab+ CT/CT(160/171) | 0.87 (0.67-1.13) |  | Keynote-042/2019[12] | Pembrolizumab/ CT (243/249) | 0.75 (0.60-0.93) |  | IMpower-110/ 2021[15] | Atezolizumab/ CT (27/23) | 0.91  (0.45-1.83) |
|  | EMPOWER-Lung3/2023 [24] | Cemiplimab+CT/ CT(38/16) | 0.60 (0.30-1.20) |  | Keynote-407/2023[14] | Pembrolizumab+ CT/CT(103/104) | 0.61 (0.45-0.83） |  | IMpower-131 /2020[17] | Atezolizumab+CT/CT (47/44) | 0.48 (0.29-0.81) |
|  | CameL-sq/ 2022[34] | Camrelizumab+CT/CT(91/97) | 0.62  (0.41-0.94) |  | Keynote-407/2023[14] | Pembrolizumab+CT/CT(73/73) | 0.68 (0.47-0.97) |  | EMPOWER-Lung1/2025[23] | Cemiplimab/CT  (123/122) | 0.51  (0.38-0.69) |
|  | AK105-302/ 2024[44] | Penpulimab+CT/ CT(59/57) | 0.69  (0.41-1.17) |  | IMpower-131/2020[17] | Atezolizumab+CT/CT(182/169) | 0.86  (0.67-1.11) |  | EMPOWER-Lung3/2023[24] | Cemiplimab+CT/CT(42/23) | 0.77  (0.40-1.45) |
|  |  |  |  |  | BFAST/2022  [22] | Atezolizumab/CT  (33/34) | 1.22  (0.67-2.21) |  | JAVELIN Lung 100/2024[26] | Avelumab/CT  (47/66) | 0.94  (0.61-1.45) |
|  |  |  |  |  | EMPOWER-Lung3/2023[24] | Cemiplimab+CT/ CT(53/28) | 0.52  (0.29-0.92) |  | CameL-sq/  2022[34] | Camrelizumab+CT/CT (37/44) | 0.48  (0.21-1.12) |
|  |  |  |  |  | EMPOWER-Lung3/2023 [24] | Cemiplimab+CT/ CT(42/23) | 0.77  (0.40-1.45) |  | AK105-302/ 2024[44] | Penpulimab+CT/CT (32/32) | 0.32  (0.14-0.73) |
|  |  |  |  |  | MYSTIC/ 2020[27] | Durvalumab/CT  (52/52) | 0.89  (0.57-1.37) |  | KEYNOTE-598/2021[47] | Pembrolizumab+ipilimumab/Pembrolizumab (77/81) | 1.16  (0.76-1.78) |
|  |  |  |  |  | PEARL/2025  [30] | Durvalumab/CT  (132/133) | 0.75  (0.58-0.98) |  |  |  |  |
|  |  |  |  |  | CameL-sq/  2022[34] | Camrelizumab+ CT/CT(95/93) | 0.52  (0.31-0.86) |  |  |  |  |
|  |  |  |  |  | AK105-302/  2024[44] | Penpulimab+CT /CT(84/85) | 0.58  (0.38-0.90) |  |  |  |  |
|  |  |  |  |  | AK105-302/  2024[44] | Penpulimab+CT /CT(32/32) | 0.32  (0.14-0.73) |  |  |  |  |
| **OS (non-SQ-**  **NSCLC)** | CheckMate- 227/2019[5] | Nivolumab+ipilimumab/CT (140/140) | 0.67  (0.51-0.88) |  | CheckMate-026/2017[4] | Nivolumab/CT  (206/206) | 1.17  (0.91-1.52) |  | CheckMate-227(part2)/  2023[6] | Nivolumab+CT/CT(68/59) | 0.56  (0.34-0.92) |
|  | CheckMate-227(part2)  /2023[6] | Nivolumab+CT/CT(113/118) | 0.91  (0.66-1.25) |  | CheckMate-227/2019[5] | Nivolumab+ipilimumab/CT(279/281) | 0.85  (0.69-1.04) |  | TASUKI-52/ 2025[9] | Nivolumab+Bev+CT /Bev+CT(73/74) | 0.70  (0.48-1.08) |
|  | CheckMate-9LA/2025[8] | Nivolumab+ipilimumab+CT/CT (99/93) | 0.69  (0.51-0.94) |  | CheckMate-227(part2)/ 2023[6] | Nivolumab+CT/CT  (137/139) | 0.76  (0.55-1.05) |  | Keynote-024/ 2019[11] | Pembrolizumab/CT(125/124) | 0.58  (0.41-0.83) |
|  | TASUKI-52/  2025[9] | Nivolumab+Bev+CT/Bev+CT (120/120) | 0.80 (0.58-1.10) |  | CheckMate-  9LA/2025[8] | Nivolumab+ipilimumab+CT/CT (130/130) | 0.78  (0.60-1.01) |  | Keynote-042/ 2019[12] | Pembrolizumab/CT (192/186) | 0.82  (0.63-1.07) |
|  | Keynote-189/2023[13] | Pembrolizumab+CT/CT(127/63) | 0.55  (0.39-0.76) |  | TASUKI-52/  2025[9] | Nivolumab+Bev+CT/Bev+CT  (82/81) | 0.60  (0.41-0.88) |  | Keynote-189/ 2023[13] | Pembrolizumab+CT/CT(132/70) | 0.68  (0.49-0.96) |
|  | IMpower130/2019[16] | Atezolizumab+ CT/CT(235/121) | 0.81  (0.61-1.08) |  | TASUKI-52/  2025[9] | Nivolumab+Bev+CT/Bev+CT  (73/74) | 0.70  (0.48-1.08) |  | IMpower-110/  2021[15] | Atezolizumab/CT(80/75) | 0.72  (0.48-1.08) |
|  | IMpower132/2021[18] | Atezolizumab+ CT/CT(88/75) | 0.67 (0.46-0.96) |  | Keynote-042/2019[12] | Pembrolizumab/ CT(394/388) | 0.86  (0.72-1.03) |  | IMpower130/  2019[16] | Atezolizumab+CT/CT(88/42) | 0.84  (0.51-1.39) |
|  | IMpower150/  2021[20] | Atezolizumab+Bev+CT/Bev+CT  (167/173) | 0.90  (0.72-1.14) |  | Keynote-189/2023[13] | Pembrolizumab+ CT/CT(128/58) | 0.65  (0.46-0.90) |  | IMpower132/ 2021[18] | Atezolizumab+CT/CT(25/20) | 0.73  (0.31-1.73) |
|  | IMpower150/  2021[20] | Atezolizumab+ CT/Bev+CT (164/173) | 0.96  (0.76-1.22) |  | Keynote-189/2023[13] | Pembrolizumab+ CT/CT(132/70) | 0.68  (0.49-0.96) |  | IMpower150/  2021[20] | Atezolizumab+Bev+CT/  Bev+CT(71/65) | 0.70  (0.46-1.08) |
|  | EMPOWER-Lung3/2023 [24] | Cemiplimab+CT/CT(57/28) | 1.26  (0.74-2.12) |  | IMpower130/2019[16] | Atezolizumab+CT/CT(128/65) | 0.70  (0.45-1.08) |  | IMpower150/2021[20] | Atezolizumab+CT/Bev+CT(63/65) | 0.76  (0.49-1.17) |
|  | RATIONALE 304/2024[31] | Tislelizumab+CT/CT(91/48) | 1.53 (0.88-2.64) |  | IMpower130/2019[16] | Atezolizumab+CT/CT(88/42) | 0.84 (0.51-1.39) |  | EMPOWER-Lung1/2025[23] | Cemiplimab/CT (161/159) | 0.66  (0.50-0.88) |
|  | CameL/2024[33] | Camrelizumab+CT/CT(49/69) | 0.84 (0.56-1.27) |  | IMpower132/2021[18] | Atezolizumab+CT/CT(63/73) | 1.18 (0.80-1.76) |  | EMPOWER-Lung3/2023[24] | Cemiplimab+CT/CT (61/26) | 0.42 (0.23-0.76) |
|  | ASTRUM-002/2025[40] | Serpluimab+CT/CT(84/68) | 0.73 (0.48-1.10) |  | IMpower132/2021[18] | Atezolizumab+CT/CT(25/20) | 0.73 (0.31-1.73) |  | JAVELIN Lung 100/2024[26] | Avelumab/CT (104/150) | 0.81 (0.60-1.10) |
|  | ORIENT-11/2022[42] | Sintilimab+CT/ CT(77/40) | 0.75 (0.48-1.19) |  | IMpower150/2021[20] | Atezolizumab+Bev+CT/Bev+CT (192/165) | 0.73 (0.57-0.94) |  | RATIONALE 304/2024[31] | Tislelizumab+CT/CT (74/36) | 0.39 (0.21-0.70) |
|  | APPLE/2024[48] | Atezolizumab+Bev+CT/Atezolizumab+CT(71/70) | 0.87 (0.57-1.32) |  | IMpower150/2021[20] | Atezolizumab+CT/Bev+CT(185/165) | 0.71 (0.55-0.91) |  | CameL/2024 [33] | Camrelizumab+CT/CT (30/20) | 0.79 (0.34-1.79) |
|  |  |  |  |  | BFAST/ 2022[22] | Atezolizumab/CT (113/112) | 0.78 (0.55-1.11) |  | ASTRUM-002/2025[40] | Serplulimab+CT/CT (41/42) | 0.49 (0.30-0.79) |
|  |  |  |  |  | EMPOWER-Lung3/2023 [24] | Cemiplimab+CT/ CT(61/33) | 0.48 (0.28-0.82) |  | KEYNOTE-598/2021[47] | Pembrolizumab+ipilimumab/Pembrolizumab (207/203) | 1.04 (0.78-1.38) |
|  |  |  |  |  | EMPOWER-Lung3/2023 [24] | Cemiplimab+CT/ CT(61/26) | 0.42 (0.23-0.76) |  | APPLE/2024 [48] | Atezolizumab+Bev+CT/Atezolizumab+CT (41/42) | 1.21 (0.66-2.24) |
|  |  |  |  |  | MYSTIC/ 2020[27] | Durvalumab/CT (111/110) | 0.70 (0.51-0.96) |  |  |  |  |
|  |  |  |  |  | PEARL/2025[30] | Durvalumab/CT (203/201) | 0.91 (0.73-1.13) |  |  |  |  |
|  |  |  |  |  | RATIONALE 304/2024[31] | Tislelizumab+CT/CT(127/63) | 0.60 (0.38-0.96) |  |  |  |  |
|  |  |  |  |  | CameL/2024[33] | Camrelizumab+ CT/CT(138/117) | 0.71 (0.52-0.96) |  |  |  |  |
|  |  |  |  |  | ASTRUM-002/2025[40] | Serplulimab+CT/ CT(64/73) | 0.70 (0.47-0.79) |  |  |  |  |
|  |  |  |  |  | ASTRUM-002/2025[40] | Serplulimab+CT/ CT(41/42) | 0.49 (0.30-0.79) |  |  |  |  |
|  |  |  |  |  | ORIENT-11/2022[42] | Sintilimab+CT/CT (181/87) | 0.56 (0.40-0.77) |  |  |  |  |
|  |  |  |  |  | APPLE/2024[48] | Atezolizumab+Bev+CT/Atezolizumab+CT(59/48) | 1.00 (0.55-1.83) |  |  |  |  |
|  |  |  |  |  | APPLE/2024[48] | Atezolizumab+Bev+CT/Atezolizumab+CT(41/42) | 1.21 (0.66-2.24) |  |  |  |  |
| **PFS (SQ-NSCLC)** | Keynote-407/2023[14] | Pembrolizumab+CT/CT(95/99) | 0.70 (0.52-0.95) |  | CheckMate-026/2017[4] | Nivolumab/CT (65/64) | 0.83 (0.54-1.26) |  | Keynote-024/ 2016[10] | Pembrolizumab/CT (29/27) | 0.35 (0.17-0.71) |
|  | IMpower-131/2020[17] | Atezolizumab+CT/CT(160/171) | 0.82 (0.65-1.04) |  | Keynote-407/2023[14] | Pembrolizumab+ CT/CT(103/104) | 0.60 (0.45-0.81） |  | Keynote-407/ 2023[14] | Pembrolizumab+CT/CT(73/73) | 0.48 (0.33-0.69) |
|  | EMPOWER-Lung3/2023 [24] | Cemiplimab+CT/CT(38/16) | 0.70 (0.37-1.32) |  | Keynote-407/2023[14] | Pembrolizumab+ CT/CT(73/73) | 0.48 (0.33-0.69) |  | IMpower-131/ 2020[17] | Atezolizumab+CT/CT (47/44) | 0.41 (0.25-0.68) |
|  | RATIONALE-307/2024 [32] | Tislelizumab+ PC/CT(47/45) | 0.57 (0.34-0.94) |  | IMpower-131/2020[17] | Atezolizumab+CT/CT(136/125) | 0.61 (0.48-0.77) |  | EMPOWER-Lung1/2025[23] | Cemiplimab/CT (123/122) | 0.44 (0.32-0.60) |
|  | RATIONALE-307/2024[32] | Tislelizumab+ nPC/CT(46/45) | 0.65 (0.40-1.06) |  | BFAST/2022[22] | Atezolizumab/CT (33/34) | 1.14 (0.68-1.92) |  | EMPOWER-Lung3/2023[24] | Cemiplimab+CT/CT (42/23) | 0.51 (0.28-0.92) |
|  | CameL-sq/ 2022[34] | Camrelizumab+CT/CT(91/97) | 0.49 (0.35-0.68) |  | EMPOWER-Lung3/2023[24] | Cemiplimab+CT/ CT(53/28) | 0.55 (0.33-0.90) |  | JAVELIN Lung 100/2024[26] | Avelumab/CT (47/66) | 0.63 (0.38-1.05) |
|  | ASTRUM- 004/2024[39] | Serplulimab+CT/CT(135/68) | 0.46 (0.31-0.67) |  | EMPOWER-Lung3/2023 [24] | Cemiplimab+CT/ CT(42/23) | 0.51 (0.28-0.92) |  | RATIONALE-307/2024[32] | Tislelizumab+PC/CT (42/41) | 0.44 (0.26-0.75) |
|  | ORIENT-12/2021[43] | Sintilimab+CT/ CT(59/63) | 0.55 (0.37-0.82) |  | RATIONALE-307/2024 [32] | Tislelizumab+PC/CT(72/72) | 0.42 (0.28-0.63) |  | RATIONALE- 307/2024[32] | Tislelizumab+nPC/CT (42/41) | 0.33 (0.18-0.59) |
|  | AK105-302/ 2024[44] | Penpulimab+CT/CT(59/57) | 0.55 (0.36-0.84) |  | RATIONALE-307/2024 [32] | Tislelizumab+nPC/CT(72/72) | 0.35  (0.23-0.54) |  | CameL-sq/2022[34] | Camrelizumab+CT/CT (37/44) | 0.30 (0.17-0.55) |
|  | TQB2450-III-12/2025[50] | Benmelstobart+CT/Tislelizumab+CT(104/107) | 0.74 (0.47-1.18) |  | CameL-sq/2022[34] | Camrelizumab+ CT/CT(95/93) | 0.34 (0.24-0.49) |  | ASTRUM-004/2024[39] | Serplulimab+CT/CT (104/53) | 0.44 (0.28-0.68) |
|  | HARMONi-6/2025[53] | Ivonescimab+ CT/Tislelizumab+CT(105/105) | 0.55 (0.37-0.82) |  | ASTRUM-004/2024[39] | Serplulimab+CT/ CT(119/58) | 0.71 (0.47-1.06) |  | ORIENT-12/2021[43] | Sintilimab+CT/CT (58/63) | 0.46 (0.30-0.70) |
|  | Pooled analysis of CheckMate 227-9LA/2025[46] | Nivolumab+ipilimumab±CT/CT (82/82) | 0.60 (0.42-0.85) |  | ASTRUM-004/2024[39] | Serplulimab+CT/ CT(104/53) | 0.44 (0.28-0.68) |  | AK105-302/2024[44] | Penpulimab+CT/CT(32/32) | 0.24 (0.13-0.45) |
|  |  |  |  |  | ORIENT-12/2021[43] | Sintilimab+CT/CT (62/52) | 0.62 (0.41-0.94) |  | KEYNOTE-598/2021[47] | Pembrolizumab+ipilimumab/Pembrolizumab(77/81) | 0.98 (0.68-1.42) |
|  |  |  |  |  | AK105-302/ 2024[44] | Penpulimab+CT/ CT(116/117) | 0.37 (0.27-0.52) |  | TQB2450-III-12/2025[50] | Benmelstobart+CT/Tislelizumab+CT(48/51) | 1.04 (0.46-2.37) |
|  |  |  |  |  | TQB2450-III-12/2025[50] | Benmelstobart+ CT/Tislelizumab+CT(126/126) | 0.47 (0.30-0.73) |  | HARMONi-6/ 2025[53] | Ivonescimab+CT/Tislelizumab+CT(49/62) | 0.71 (0.37-1.33) |
|  |  |  |  |  | TQB2450-III-12/2025[50] | Benmelstobart+ CT/Tislelizumab+ CT(48/51) | 1.04 (0.46-2.37) |  |  |  |  |
|  |  |  |  |  | HARMONi-6/2025[53] | Ivonescimab+CT/Tislelizumab+CT (161/161) | 0.66 (0.46-0.95) |  |  |  |  |
| **PFS (non-SQ-NSCLC)** | TASUKI-52/2025[9] | Nivolumab+Bev+CT/Bev+CT (120/120) | 0.63 (0.45-0.87) |  | CheckMate-026/2017[4] | Nivolumab/CT(206/206) | 1.29(1.02-1.63) |  | TASUKI-52/ 2025[9] | Nivolumab+Bev+CT/Bev+CT(73/74) | 0.49 (0.33-0.73) |
|  | Keynote-189/2023[13] | Pembrolizumab+CT/CT(127/63) | 0.67 (0.49-0.92) |  | TASUKI-52/ 2025[9] | Nivolumab+Bev+CT/Bev+CT (82/81) | 0.66 (0.44-0.99) |  | Keynote-024/ 2016[10] | Pembrolizumab/CT (125/124) | 0.55 (0.39-0.76) |
|  | IMpower130/2019[16] | Atezolizumab+ CT/CT(235/121) | 0.72 (0.56-0.91) |  | TASUKI-52/ 2025[9] | Nivolumab+Bev+CT/Bev+CT  (73/74) | 0.49 (0.33-0.73) |  | Keynote-189/ 2023[13] | Pembrolizumab+CT/CT(132/70) | 0.35 (0.25-0.49) |
|  | IMpower132/2021[18] | Atezolizumab+ CT/CT(88/75) | 0.45 (0.31-0.64) |  | Keynote-189/2023[13] | Pembrolizumab+ CT/CT(128/58) | 0.57 (0.41-0.80) |  | IMpower130/ 2019[16] | Atezolizumab+CT/CT (88/42) | 0.51 (0.34-0.77) |
|  | IMpower150/2018[19] | Atezolizumab+Bev+CT/Bev+CT (166/167) | 0.77 (0.61-0.92) |  | Keynote-189/2023[13] | Pembrolizumab+ CT/CT(132/70) | 0.35 (0.25-0.49) |  | IMpower132/ 2021[18] | Atezolizumab+CT/CT (25/20) | 0.46 (0.22-0.96) |
|  | EMPOWER-Lung3/2023  [24] | Cemiplimab+CT/CT(57/28) | 0.79  (0.49-1.30) |  | IMpower130/2019[16] | Atezolizumab+CT/CT(128/65) | 0.61  (0.43-0.85) |  | IMpower150/2018[19] | Atezolizumab+Bev+CT/Bev+CT(71/64) | 0.39(0.25-0.60) |
|  | RATIONALE 304/2024[31] | Tislelizumab+CT/CT(91/48) | 0.83  (0.53-1.28) |  | IMpower130/2019[16] | Atezolizumab+CT/CT(88/42) | 0.51  (0.34-0.77) |  | EMPOWER-Lung1/2025[23] | Cemiplimab/CT  (161/159) | 0.55  (0.42-0.72) |
|  | CameL/2024[33] | Camrelizumab+CT/CT(49/69) | 0.75  (0.50-1.13) |  | IMpower132/2021[18] | Atezolizumab+CT/CT(63/73) | 0.80  (0.56-1.16) |  | EMPOWER-Lung3/2023[24] | Cemiplimab+CT/CT  (61/26) | 0.46  (0.27-0.80) |
|  | ASTRUM-002/2025[40] | Serpluimab+CT/CT(84/68) | 0.82  (0.53-1.26) |  | IMpower132/2021[18] | Atezolizumab+CT/CT(25/20) | 0.46  (0.22-0.96) |  | JAVELIN Lung 100/2024[26] | Avelumab/CT  (104/150) | 0.74  (0.54-1.02) |
|  | ORIENT-11/2021[41] | Sintilimab+CT/  CT(85/44) | 0.60  (0.39-0.92) |  | IMpower150/2018[19] | Atezolizumab+  Bev+CT/Bev+CT  (119/105) | 0.56  (0.41-0.77) |  | RATIONALE 304/2024[31] | Tislelizumab+CT/CT  (74/36) | 0.29  (0.16-0.50) |
|  | APPLE/2024[48] | Atezolizumab+Bev+CT/Atezolizumab+CT(71/70) | 1.11  (0.78-1.56) |  | IMpower150/2018[19] | Atezolizumab+  Bev+CT/Bev+CT  (71/64) | 0.39  (0.25-0.60) |  | CameL/2024[33] | Camrelizumab+CT/CT  (30/20) | 0.41  (0.19-0.85) |
|  | Pooled analysis of CheckMate 227-9LA/2025[46] | Nivolumab+ipilimumab±CT/CT  (240/233) | 0.77  (0.63-0.95) |  | BFAST/2022[22] | Atezolizumab/CT  (113/112) | 0.65  (0.48-0.88) |  | ASTRUM-002/2025[40] | Serplulimab+CT/CT  (62/62) | 0.40  (0.25-0.63) |
|  |  |  |  |  | EMPOWER-Lung3/2023  [24] | Cemiplimab+CT/  CT(61/33) | 0.42  (0.26-0.69) |  | KEYNOTE-598/2021[47] | Pembrolizumab+ipilimumab/Pembrolizumab  (207/203) | 1.12  (0.88-1.43) |
|  |  |  |  |  | EMPOWER-Lung3/2023  [24] | Cemiplimab+CT/  CT(61/26) | 0.46  (0.27-0.80) |  | APPLE/2024  [48] | Atezolizumab+Bev+CT/Atezolizumab+CT  (41/42) | 0.94  (0.57-1.55) |
|  |  |  |  |  | RATIONALE 304/2024[31] | Tislelizumab+CT/CT(127/63) | 0.48  (0.32-0.72) |  |  |  |  |
|  |  |  |  |  | CameL/2024[33] | Camrelizumab+  CT/CT(138/117) | 0.51  (0.38-0.68) |  |  |  |  |
|  |  |  |  |  | ASTRUM-002/2025[40] | Serplulimab+CT/  CT(64/73) | 0.59  (0.38-0.88) |  |  |  |  |
|  |  |  |  |  | ASTRUM-002/2025[40] | Serplulimab+CT/  CT(62/62) | 0.40  (0.25-0.63) |  |  |  |  |
|  |  |  |  |  | ORIENT-11/2021[41] | Sintilimab+CT/CT  (181/87) | 0.42  (0.31-0.56) |  |  |  |  |
|  |  |  |  |  | APPLE/2024[48] | Atezolizumab+  Bev+CT/Atezolizumab+CT(59/48) | 0.82  (0.54-1.24) |  |  |  |  |
|  |  |  |  |  | APPLE/2024[48] | Atezolizumab+  Bev+CT/Atezolizumab+CT(41/42) | 0.94  (0.57-1.55) |  |  |  |  |

OS, overall survival; PFS, progression-free survival; HR, hazard ratio; CI, confidence interval; SCC, squamous cell carcinoma; NSCLC, non-small cell lung cancer; CT, chemotherapy; Bev, bevacizumab; PC, paclitaxel plus carboplatin; nPC, nab-paclitaxel plus carboplatin.

**Table S3** Comparison of the fit goodness between consistency and inconsistency models based on DIC values.

| **Outcome** | **Model** | **DIC** | **I^2^(%)** |
| --- | --- | --- | --- |
| Overall survival (SQ-NSCLC) | Consistency | 50.23 | 0 |
|  | Inconsistency | 51.83 | 0 |
| Overall survival (non-SQ-NSCLC) | Consistency | 56.75 | 2 |
|  | Inconsistency | 60.00 | 6 |
| Progression-free survival (SQ-NSCLC) | Consistency | 46.09 | 0 |
|  | Inconsistency | 47.95 | 4 |
| Progression-free survival (non-SQ-NSCLC) | Consistency | 46.02 | 4 |
|  | Inconsistency | 46.29 | 1 |
| Grade 3-5 TRAEs | Consistency | 158.89 | 19 |
|  | Inconsistency | 159.03 | 19 |

The DIC provides a measure of model fit adjusted with the complexity of the model, with lower values correspond to preferable models and differences of 5 considered important.

DIC, Deviance information criterion; SQ-NSCLC, squamous non-small cell lung cancer; TRAEs, treatment-related adverse events.

**Table S4** Comparison of outcomes derived from pairwise and network meta-analyses

| **Comparison** | **HR/OR (95% CI)** | **p-value*** |
| --- | --- | --- |
| **Overall survival (SQ-NSCLC)** | | |
| Atezolizumab vs CT | | 0.998 |
| Pairwise (Frequentist) | 0.89(0.68-1.16) |  |
| Pairwise (Bayesian) | 0.90(0.68-1.16) |  |
| Network (Bayesian) | 0.89(0.68-1.16) |  |
| Durvalumab vs CT | | 0.996 |
| Pairwise (Frequentist) | 0.78(0.63-0.98) |  |
| Pairwise (Bayesian) | 0.79(0.63-0.98) |  |
| Network (Bayesian) | 0.78(0.63-0.98) |  |
| Pembrolizumab vs CT | | 0.994 |
| Pairwise (Frequentist) | 0.75(0.61-0.92) |  |
| Pairwise (Bayesian) | 0.75(0.61-0.92) |  |
| Network (Bayesian) | 0.74(0.61-0.90) |  |
| **Overall survival (non-SQ-NSCLC)** | | |
| Atezolizumab vs CT | | 1.000 |
| Pairwise (Frequentist) | 0.76(0.63-0.92) |  |
| Pairwise (Bayesian) | 0.76(0.63-0.92) |  |
| Network (Bayesian) | 0.76(0.63-0.93) |  |
| Atezolizumab + CT vs CT | | 0.729 |
| Pairwise (Frequentist) | 0.76(0.63-0.92) |  |
| Pairwise (Bayesian) | 0.83(0.71-0.96) |  |
| Network (Bayesian) | 0.83(0.71-0.96) |  |
| Durvalumab vs CT | | 0.993 |
| Pairwise (Frequentist) | 0.84(0.70-1.00) |  |
| Pairwise (Bayesian) | 0.83(0.71-0.95) |  |
| Network (Bayesian) | 0.84(0.70-1.00) |  |
| Pembrolizumab vs CT | | 0.909 |
| Pairwise (Frequentist) | 0.73(0.50-1.07) |  |
| Pairwise (Bayesian) | 0.80(0.68-0.93) |  |
| Network (Bayesian) | 0.79(0.68-0.93) |  |
| **Progression-free survival (non-SQ-NSCLC)** | | |
| Atezolizumab + CT vs CT | | 1.000 |
| Pairwise (Frequentist) | 0.62(0.55-0.71) |  |
| Pairwise (Bayesian) | 0.62(0.54-0.71) |  |
| Network (Bayesian) | 0.62(0.54-0.71) |  |
| **Grade 3-5 treatment-related adverse events** | | |
| Atezolizumab vs CT | | 1.000 |
| Pairwise (Frequentist) | 0.26(0.20-0.33) |  |
| Pairwise (Bayesian) | 0.26(0.20-0.33) |  |
| Network (Bayesian) | 0.26(0.20-0.33) |  |
| Atezolizumab + CT vs CT | | 0.999 |
| Pairwise (Frequentist) | 1.78(1.47-2.15) |  |
| Pairwise (Bayesian) | 1.79(1.48-2.15) |  |
| Network (Bayesian) | 1.78(1.47-2.16) |  |
| Camrelizumab+CT | | 0.991 |
| Pairwise (Frequentist) | 1.66(0.71-3.89) |  |
| Pairwise (Bayesian) | 1.81(0.63-4.15) |  |
| Network (Bayesian) | 1.73(1.28-2.34) |  |
| Durvalumab vs CT | | 1.000 |
| Pairwise (Frequentist) | 0.27(0.17-0.43) |  |
| Pairwise (Bayesian) | 0.27(0.17-0.35) |  |
| Network (Bayesian) | 0.27(0.21-0.35) |  |
| Pembrolizumab vs CT | | 1.000 |
| Pairwise (Frequentist) | 0.31(0.25-0.39) |  |
| Pairwise (Bayesian) | 0.31(0.25-0.39) |  |
| Network (Bayesian) | 0.31(0.25-0.39) |  |
| Pembrolizumab + CT vs CT | | 0.712 |
| Pairwise (Frequentist) | 1.87(0.56-6.28) |  |
| Pairwise (Bayesian) | 2.01(0.64-5.01) |  |
| Network (Bayesian) | 1.37(1.00-1.87) |  |

*For comparison among the corresponding three groups. Only comparisons involving two or more trials are presented here.

HR, hazard ratio; OR, odds ratio; CI, confidence interval; SQ-NSCLC, squamous non-small cell lung cancer; CT, chemotherapy.

**Table S5** Node-Splitting Analysis for Assessing Local Inconsistency.

| **Nodes** | **Direct effect** | **Indirect effect** | **Overall** | **P value** |
| --- | --- | --- | --- | --- |
| **Overall survival (SQ-NSCLC)** | | | | |
| Niv+Ipi+CT vs CT | 0.65 (0.49, 0.86) | 0.76 (0.34, 1.70) | 0.66 (0.51, 0.86) | 0.71 |
| Pem vs CT | 0.75 (0.61, 0.92) | 0.64 (0.28, 1.40) | 0.74 (0.61, 0.91) | 0.71 |
| Pem vs Niv+Ipi+CT | 0.98 (0.45, 2.10) | 1.10 (0.81, 1.60) | 1.10 (0.82, 1.50) | 0.71 |
| **Overall survival (non-SQ-NSCLC)** | | | | |
| Niv+Ipi+CT vs CT | 0.79 (0.65, 0.96) | 0.64 (0.41, 1.00) | 0.76 (0.64, 0.91) | 0.40 |
| Pem+CT vs CT | 0.60 (0.50, 0.72) | 0.74 (0.47, 1.20) | 0.62 (0.52, 0.73) | 0.40 |
| Pem+CT vs Niv+Ipi+CT | 0.94 (0.62, 1.40) | 0.76 (0.58, 0.99) | 0.81 (0.65, 1.00) | 0.40 |
| **Progression-free survival (SQ-NSCLC)** | | | | |
| Niv+Ipi+CT vs CT | 0.57 (0.42, 0.78) | 0.52 (0.28, 0.96) | 0.56 (0.42, 0.74) | 0.80 |
| Pem+CT vs CT | 0.62 (0.52, 0.74) | 0.68 (0.35, 1.30) | 0.62 (0.52, 0.74) | 0.80 |
| Pem+CT vs Niv+Ipi+CT | 1.20 (0.66, 2.10) | 1.10 (0.76, 1.60) | 1.10 (0.82, 1.50) | 0.79 |
| **Progression-free survival (non-SQ-NSCLC)** | | | | |
| Niv+Ipi+CT vs CT | 0.74 (0.60, 0.91) | 0.56 (0.39, 0.79) | 0.68 (0.57, 0.82) | 0.18 |
| Pem+CT vs CT | 0.50 (0.42, 0.60) | 0.67 (0.46, 0.97) | 0.53 (0.45, 0.62) | 0.18 |
| Pem+CT vs Niv+Ipi+CT | 0.90 (0.66, 1.20) | 0.67 (0.51, 0.89) | 0.77 (0.63, 0.95) | 0.17 |

Significant inconsistency between direct and indirect evidence is indicated by P < 0.05.

SQ-NSCLC, squamous non-small cell lung cancer; Niv, Nivolumab; Ipi, Ipilimumab; Pem, pembrolizumab; CT, chemotherapy.

**TableS6** Heterogeneity assessment for comparisons informed by two or more trials

| Outcome | Treatment | No. of  studies | No. of  patients | HR (95%CI) | Heterogeneity | |
| --- | --- | --- | --- | --- | --- | --- |
|  |  |  |  |  | I^2^ | P value |
| OS (SQ-NSCLC) | Atezolizumab vs CT | 3 | 310 | 0.89(0.68-1.16) | 0 | 0.48 |
|  | Durvalumab vs CT | 2 | 369 | 0.78(0.63-0.98) | 0 | 0.51 |
|  | Pembrolizumab vs CT | 2 | 598 | 0.75(0.61-0.92) | 0 | 0.94 |
| OS (non-SQ-NSCLC) | Atezolizuma vs CT | 3 | 640 | 0.76(0.63-0.92) | 0 | 0.95 |
|  | Atezolizuma+CT vs CT | 2 | 1257 | 0.83(0.71-0.96) | 0 | 0.57 |
|  | Durvalumab vs CT | 2 | 625 | 0.84(0.70-1.00) | 44% | 0.18 |
|  | Pembrolizumab vs CT | 2 | 1031 | 0.73(0.50-1.07) | 74% | 0.05 |
| PFS (non-SQ-NSCLC) | Atezolizuma vs CT | 2 | 127 | 0.62(0.55-0.71) | 0 | 0.63 |

Abbreviations: Significant results are in bold and underlined. No., number; HR, hazard ratio; CI, confidence interval; SQ-NSCLC, squamous non-small cell lung cancer; OS, overall survival; CT, chemotherapy; PFS, progression-free survival.

**Table S7** Previous meta-analyses assessing efficacy of ICIs in advanced SQ-NSCLC and non-SQ-NSCLC

| First author  /Published year | No. of trials | No. of ICI treatment | Histology | Conclusion |
| --- | --- | --- | --- | --- |
|  |  |  |  |  |
| Shao/2022[72] | 11 | 12 | non-SQ-NSCLC | ICI combination showed better survival benefits than chemotherapy. Pembrolizumab + chemotherapy could provide the best OS benefits, whereas atezolizumab + bevacizumab + chemotherapy could bring the best PFS benefits. |
| Chen/2023[73] | 11 | 12 | non-SQ-NSCLC | For patients with PD-L1≥50%, pembrolizumab plus chemotherapy, tislelizumab plus chemotherapy, and sintilimab plus chemotherapy are recommended as good treatment options. |
| Molife/2023[74] | 11 | 9 | non-SQ-NSCLC | Sintilimab + pemetrexed + platinum showed comparable efficacy and safety versus US standard-of-care first-line ICI combinations. |
| Fedyanin/2026[75] | 15 | 15 | non-SQ-NSCLC | Irrespective of PD-L1 expression, prolgolimab, pembrolizumab or cemiplimab, each combined with chemotherapy, are most efficacious by OS; nivolumab or atezolizumab combined with bevacizumab and chemotherapy demonstrate the highest PFS. |
| Chen/2024[76] | 9 | 9 | SQ-NSCLC | For patients with PD-L1 expression ≥50%, the first-line camrelizumab plus chemotherapy provides superior OS and PFS outcomes. |
| Liu/2025[77] | 9 | 9 | SQ-NSCLC | When combined with first-line chemotherapy, camrelizumab has the potential to be a preferred option. |
| Liu/2025[78] | 25 | 20 | SQ-NSCLC | It is important to emphasize that clinical decisions should be made in the context of individual patient characteristics, including comorbidities, drug accessibility, and tolerability. |

ICI, immune checkpoint inhibitor; OS, overall survival; PFS, progression-free survival; SQ-NSCLC, squamous non-small cell lung cancer.

**
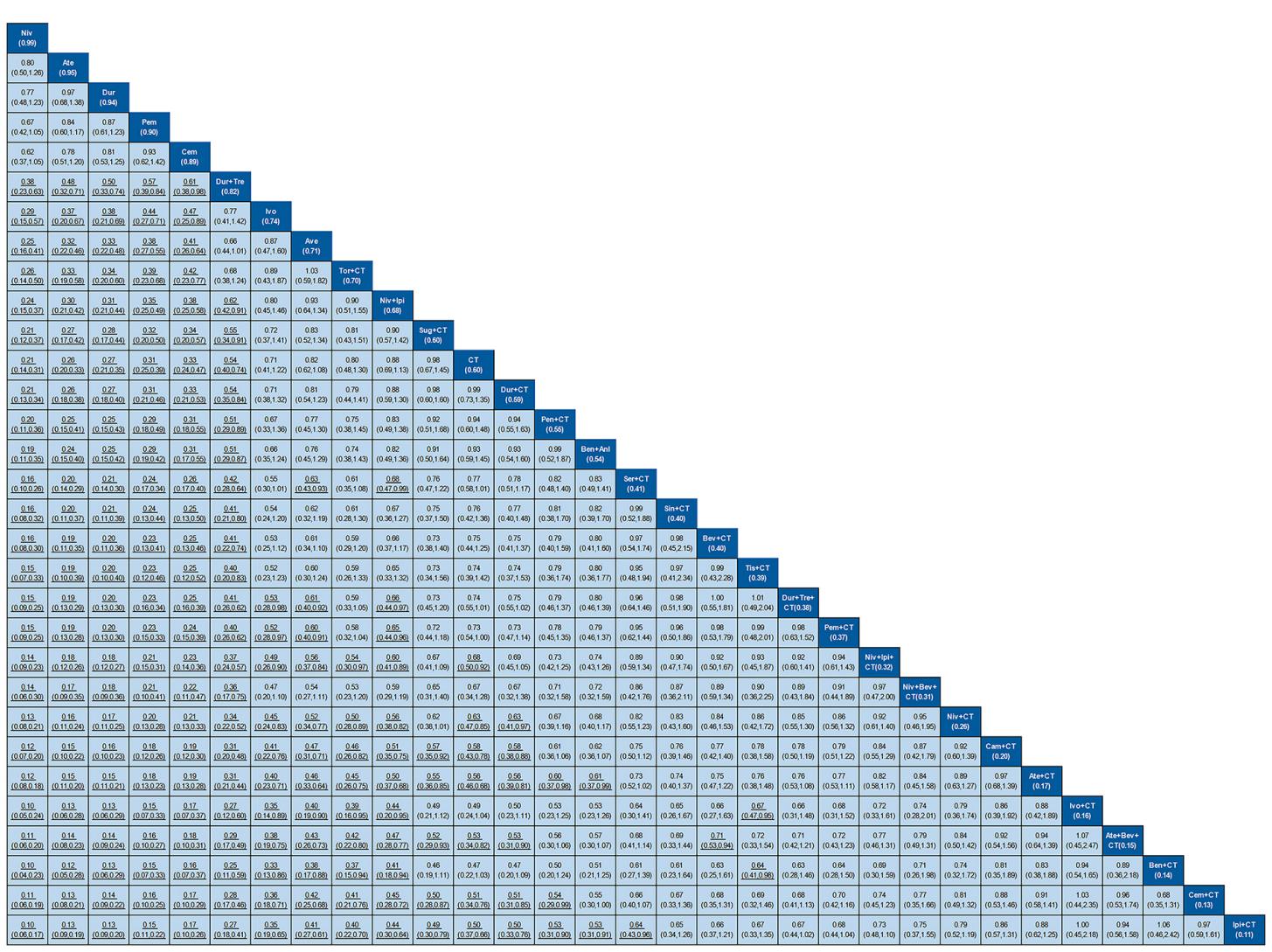
**

**Figure S1** Pooled grade 3-5 TRAEs estimates of multiple comparisons in network meta-analysis. Treatment regimens are presented in order of risk of grade 3-5 TRAEs ranking from low to high according to SUCRAs. Dark blue represents the treatment regimens (SUCRA values). Data are ORs (95% CIs) for column-defined treatment versus row-defined treatment. Significant results are underlined.

TRAEs, treatment-related adverse events; Anl, anlotinib; Ate, atezolizumab; Ave, avelumab; Ben, benmelstobart; Bev, bevacizumab; Cam, camrelizumab; Cem, cemiplimab; Dur, durvalumab; Ipi, ipilimumab; Ivo, ivonescimab; Niv, nivolumab; Pem, pembrolizumab; Pen, penpulimab; Ser, serplulimab; Sin, sintilimab; Sug, sugemalimab; Tis, tislelizumab; Tor, toripalimab; Tre, tremelimumab; CT, chemotherapy.

**
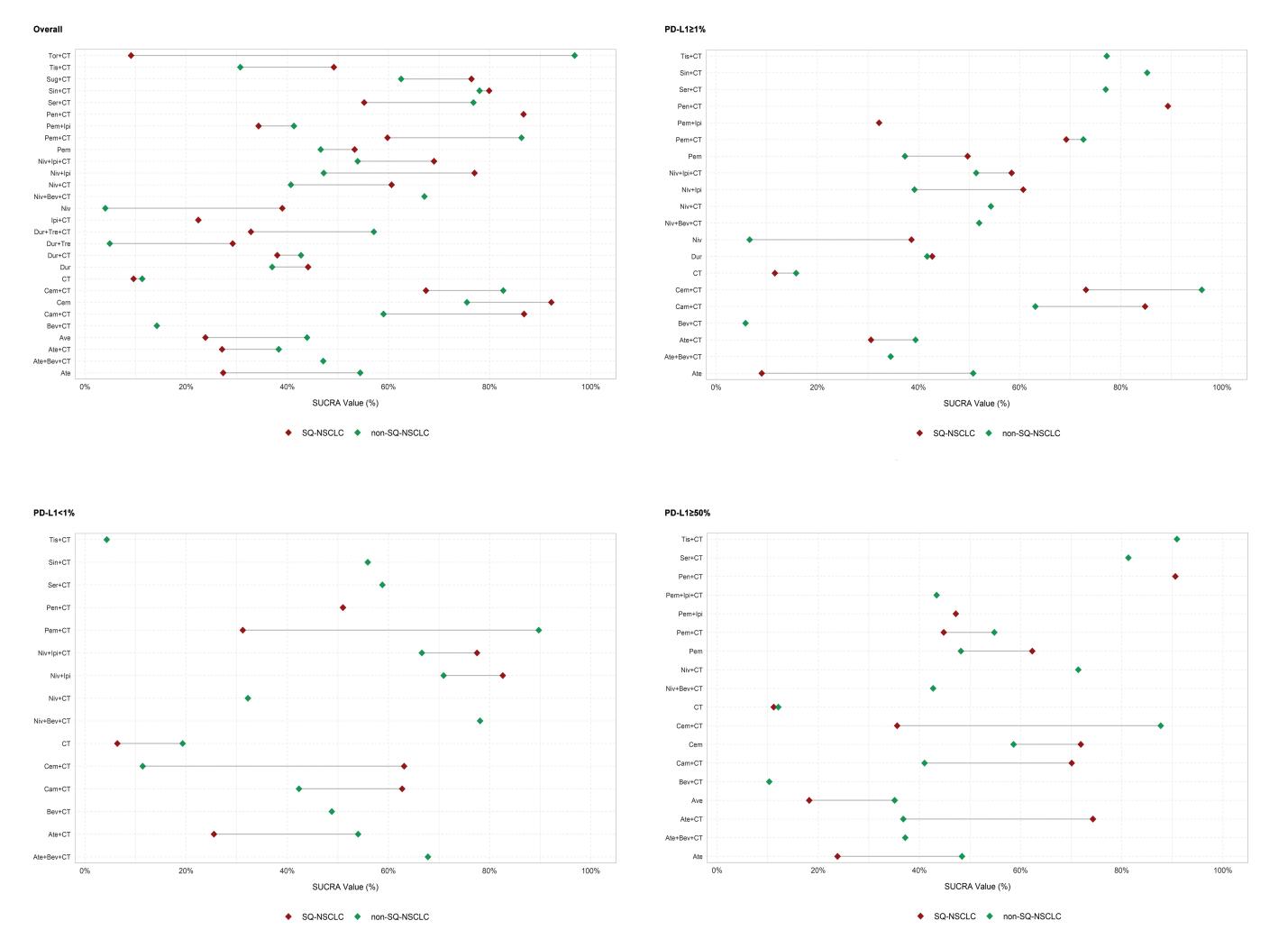
**

**Figure S2** SUCRA values of individual regimens for overall survival. SUCRA, surface under the cumulative ranking; SQ-NSCLC, squamous non-small cell lung cancer; Anl, anlotinib; Ate, atezolizumab; Ave, avelumab; Ben, benmelstobart; Bev, bevacizumab; Cam, camrelizumab; Cem, cemiplimab; Dur, durvalumab; Ipi, ipilimumab; Ivo, ivonescimab; Niv, nivolumab; Pem, pembrolizumab; Pen, penpulimab; Ser, serplulimab; Sin, sintilimab; Sug, sugemalimab; Tis, tislelizumab; Tor, toripalimab; Tre, tremelimumab; CT, chemotherapy.

**
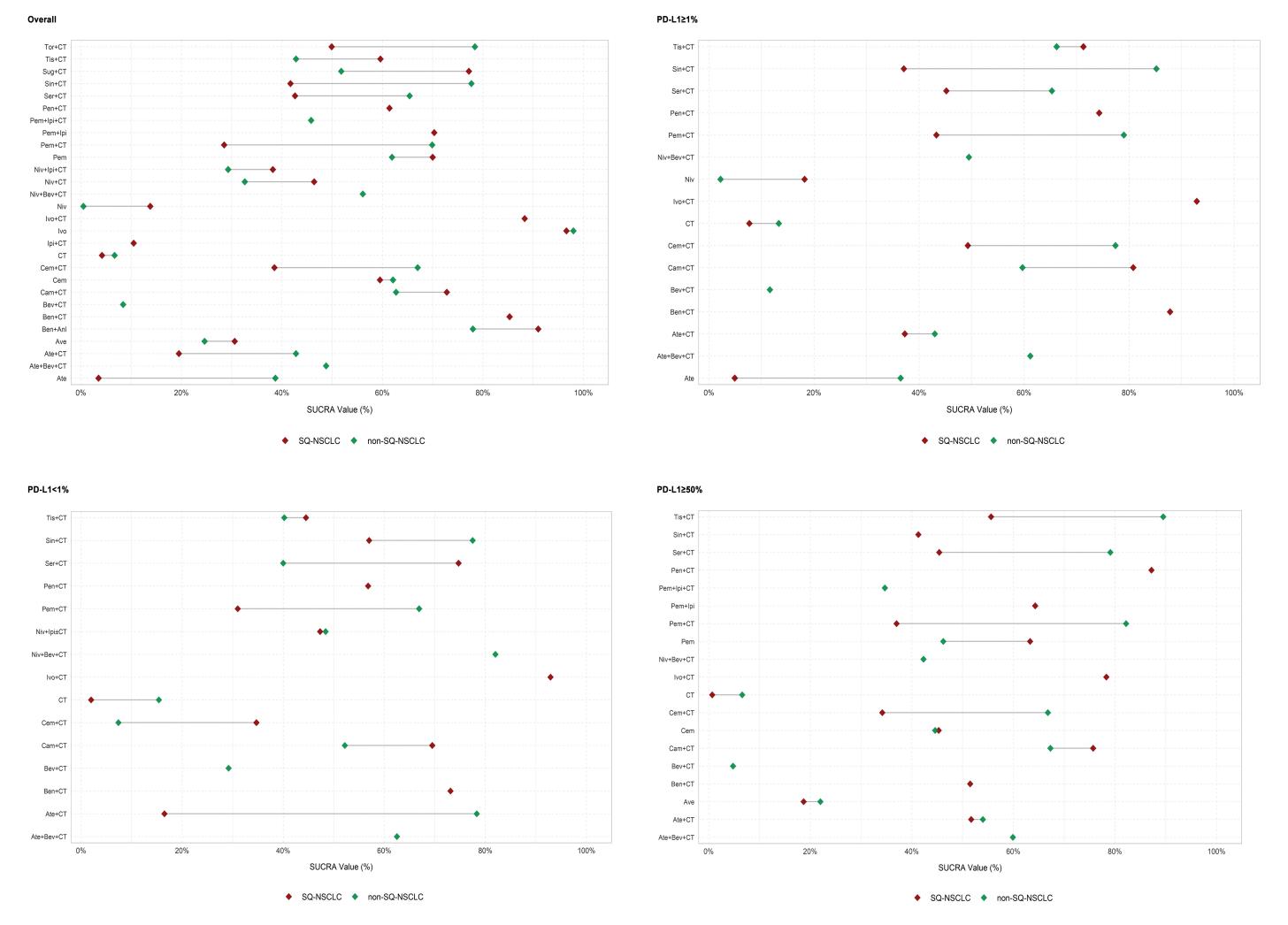
**

**Figure S3** SUCRA values of individual regimens for progression-free survival. SUCRA, surface under the cumulative ranking; SQ-NSCLC, squamous non-small cell lung cancer; Anl, anlotinib; Ate, atezolizumab; Ave, avelumab; Ben, benmelstobart; Bev, bevacizumab; Cam, camrelizumab; Cem, cemiplimab; Dur, durvalumab; Ipi, ipilimumab; Ivo, ivonescimab; Niv, nivolumab; Pem, pembrolizumab; Pen, penpulimab; Ser, serplulimab; Sin, sintilimab; Sug, sugemalimab; Tis, tislelizumab; Tor, toripalimab; Tre, tremelimumab; CT, chemotherapy.

**
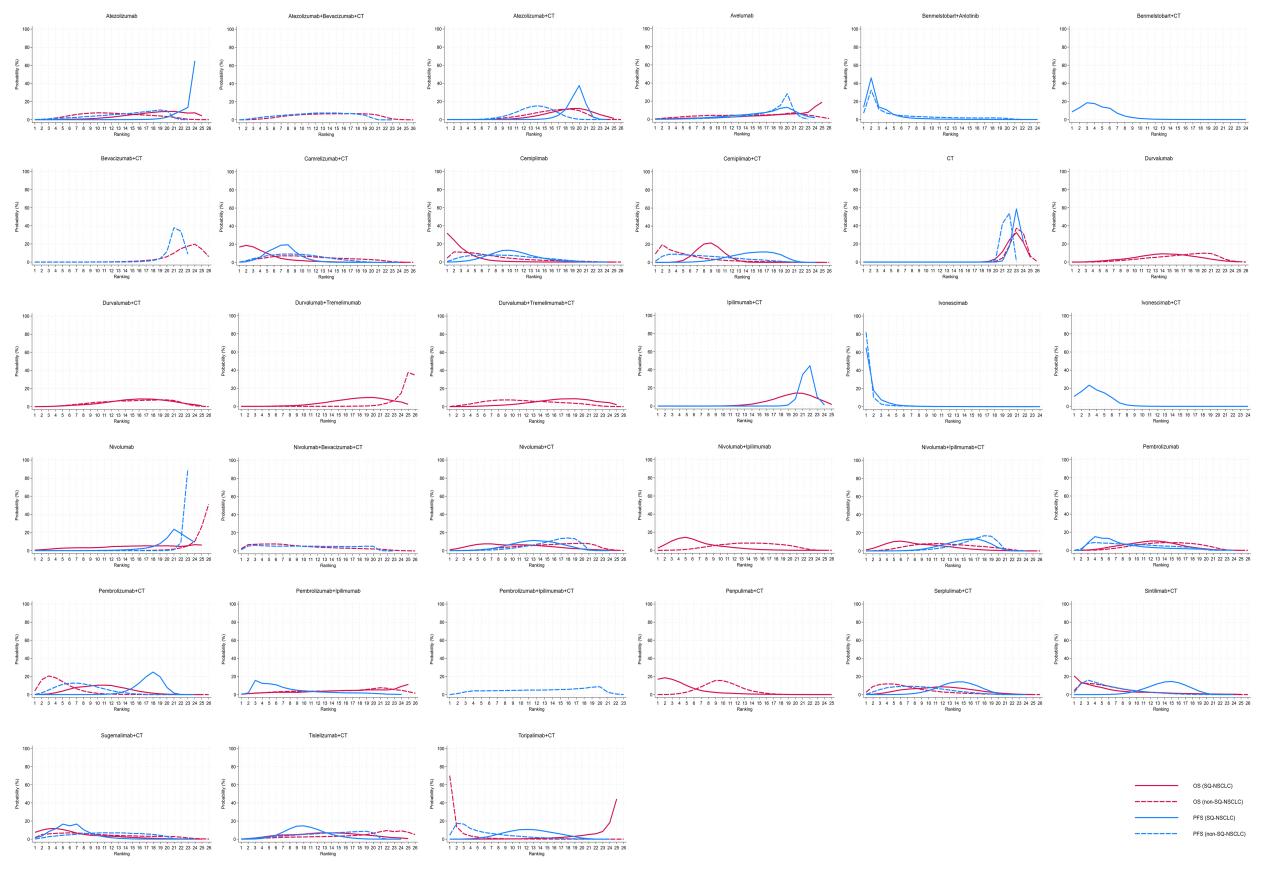
**

**Figure S4** Ranking curves indicating the probability of each comparable treatment being ranked from first to last on efficacy. OS, overall survival; PFS, progression-free survival; SQ-NSCLC, squamous non-small cell lung cancer; CT, chemotherapy.

**
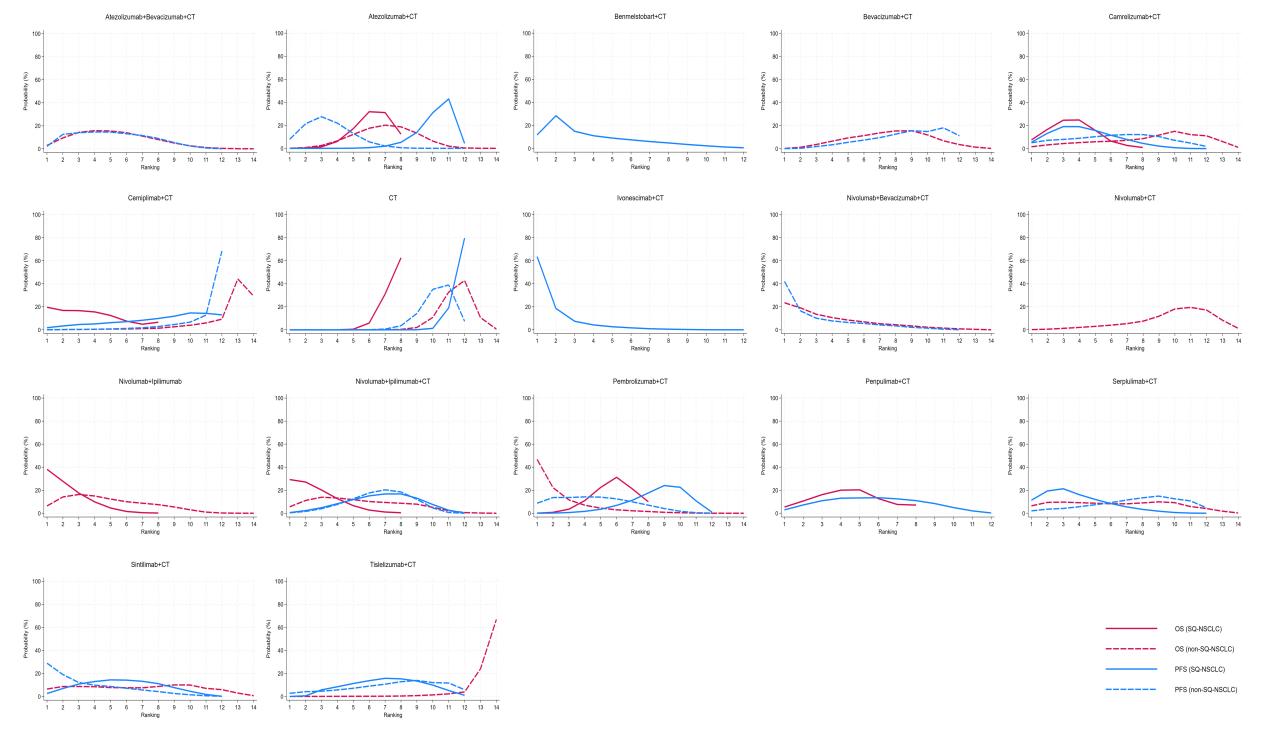
**

**Figure S5** Ranking curves indicating the probability of each comparable treatment being ranked from first to last on efficacy for subgroup of PD-L1 < 1%. OS, overall survival; PFS, progression-free survival; SQ-NSCLC, squamous non-small cell lung cancer; CT, chemotherapy.

**
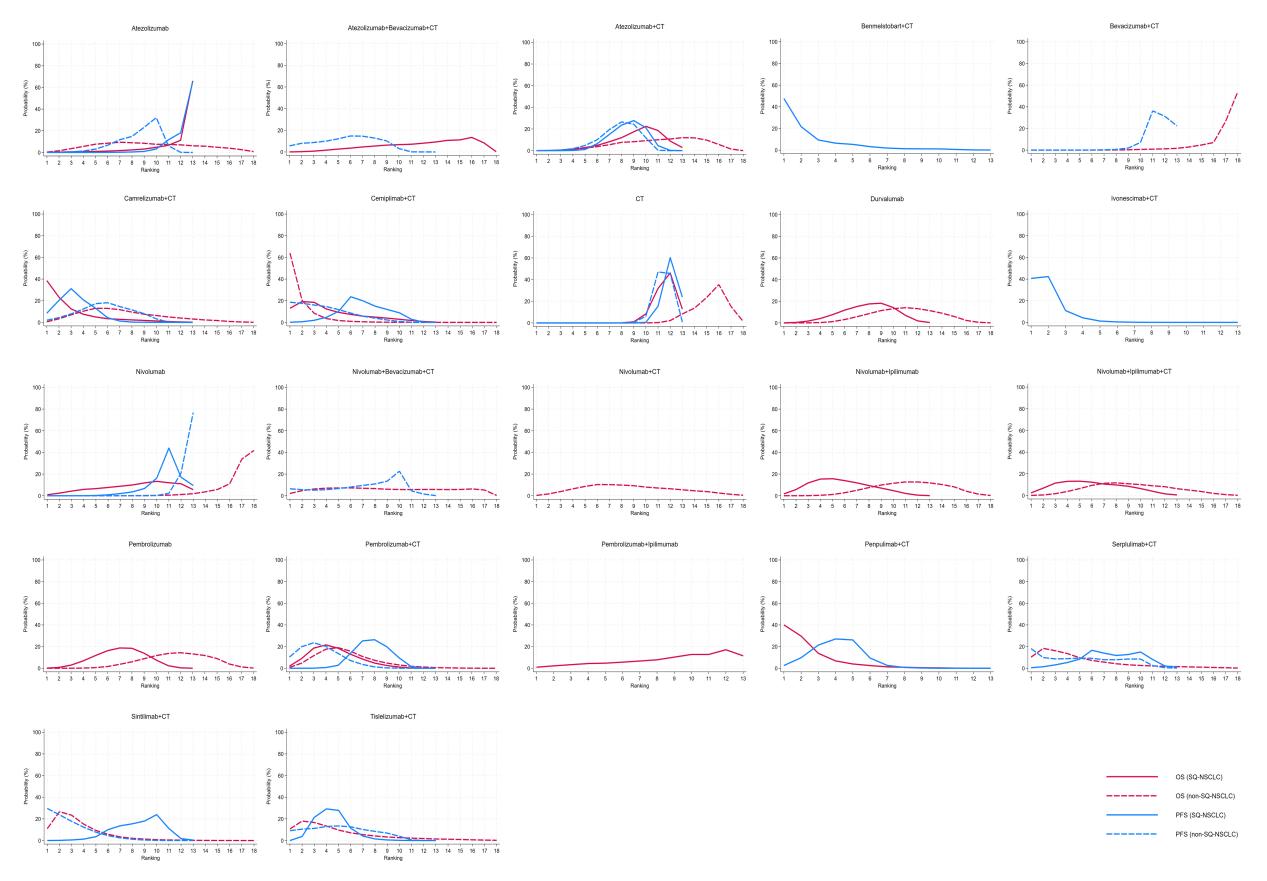
**

**Figure S6** Ranking curves indicating the probability of each comparable treatment being ranked from first to last on efficacy for subgroup of PD-L1 ≥ 1%. OS, overall survival; PFS, progression-free survival; SQ-NSCLC, squamous non-small cell lung cancer; CT, chemotherapy.

**
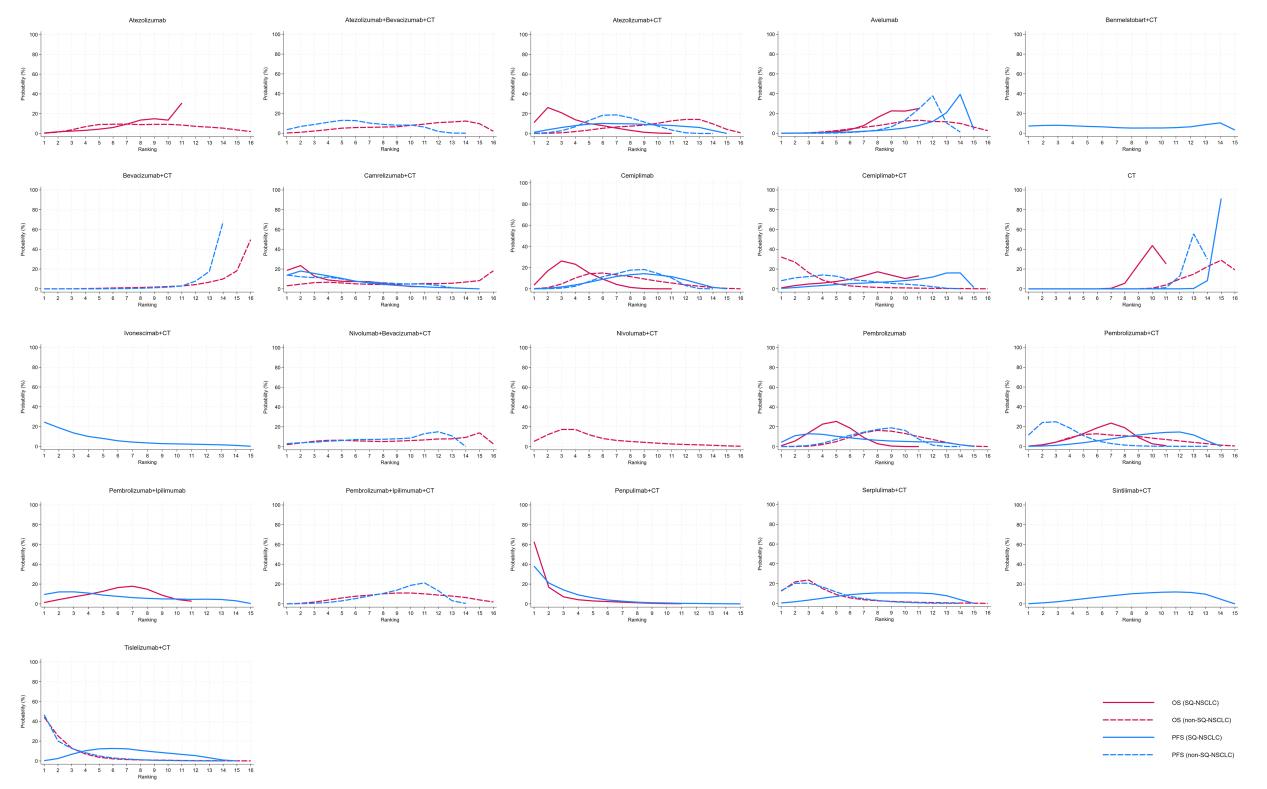
**

**Figure S7** Ranking curves indicating the probability of each comparable treatment being ranked from first to last on efficacy for subgroup of PD-L1 ≥ 50%. OS, overall survival; PFS, progression-free survival; SQ-NSCLC, squamous non-small cell lung cancer; CT, chemotherapy.

**
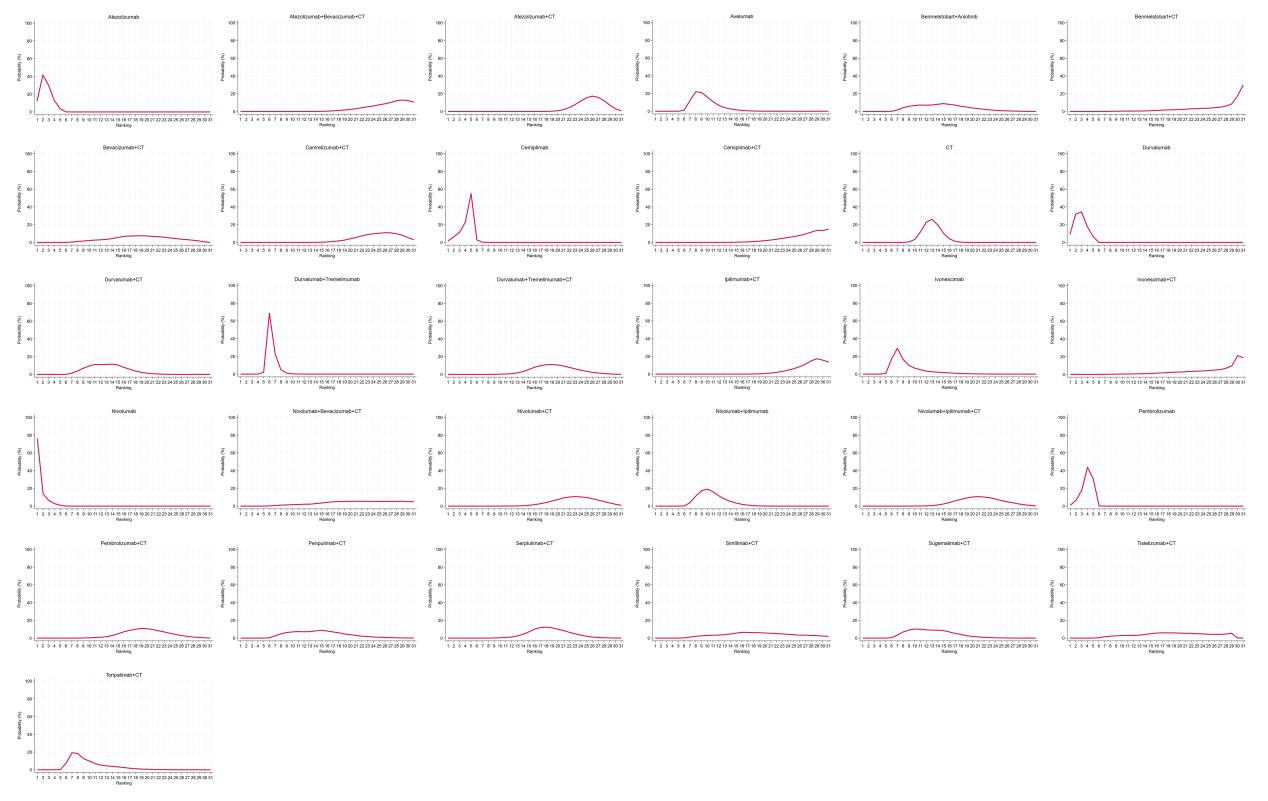
**

**Figure S8** Ranking curves indicating the probability of each comparable treatment being ranked from least to highest risk of grade 3–5 TRAEs. TRAEs, treatment-related adverse events; CT, chemotherapy.


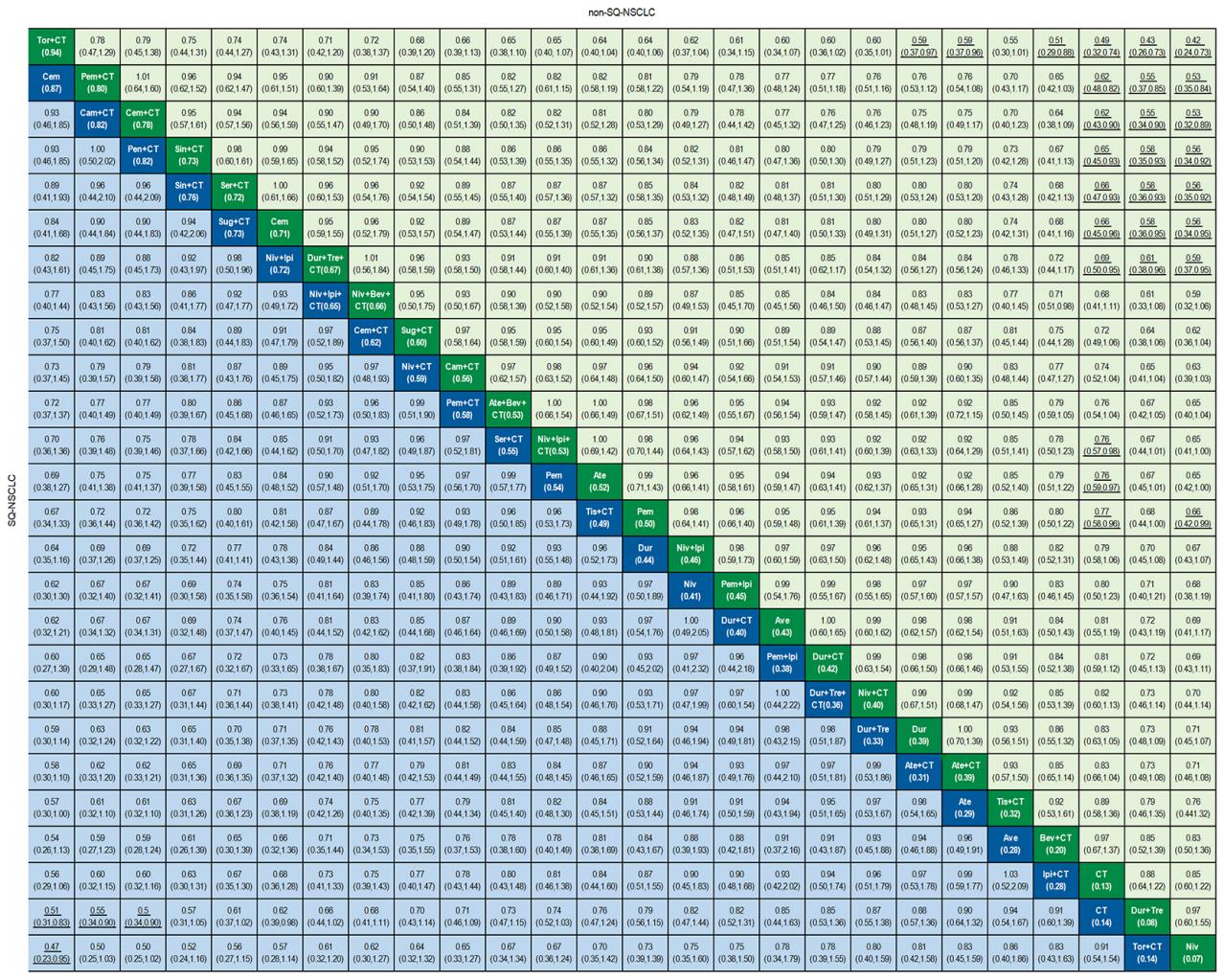


**Figure S9** Sensitivity analysis for OS using a random‑effects model. Treatment regimens are presented in order of OS ranking from high to low according to SUCRAs. Dark blue represents the regimens for SQ-NSCLC (SUCRA values), and dark green represents the regimens for non-SQ-NSCLC (SUCRA values). Data are HRs (95% CrIs) for column-defined treatment versus row-defined treatment for SQ-NSCLC (lower triangle) and row-defined treatment versus column-defined treatment for non-SQ-NSCLC (upper triangle). Significant results are underlined.

OS, overall survival; SUCRA, surface under the cumulative ranking; HR, hazard ratio; CI, confidence interval; SQ-NSCLC, squamous non-small cell lung cancer; Anl, anlotinib; Ate, atezolizumab; Ave, avelumab; Ben, benmelstobart; Bev, bevacizumab; Cam, camrelizumab; Cem, cemiplimab; Dur, durvalumab; Ipi, ipilimumab; Ivo, ivonescimab; Niv, nivolumab; Pem, pembrolizumab; Pen, penpulimab; Ser, serplulimab; Sin, sintilimab; Sug, sugemalimab; Tis, tislelizumab; Tor, toripalimab; Tre, tremelimumab; CT, chemotherapy.


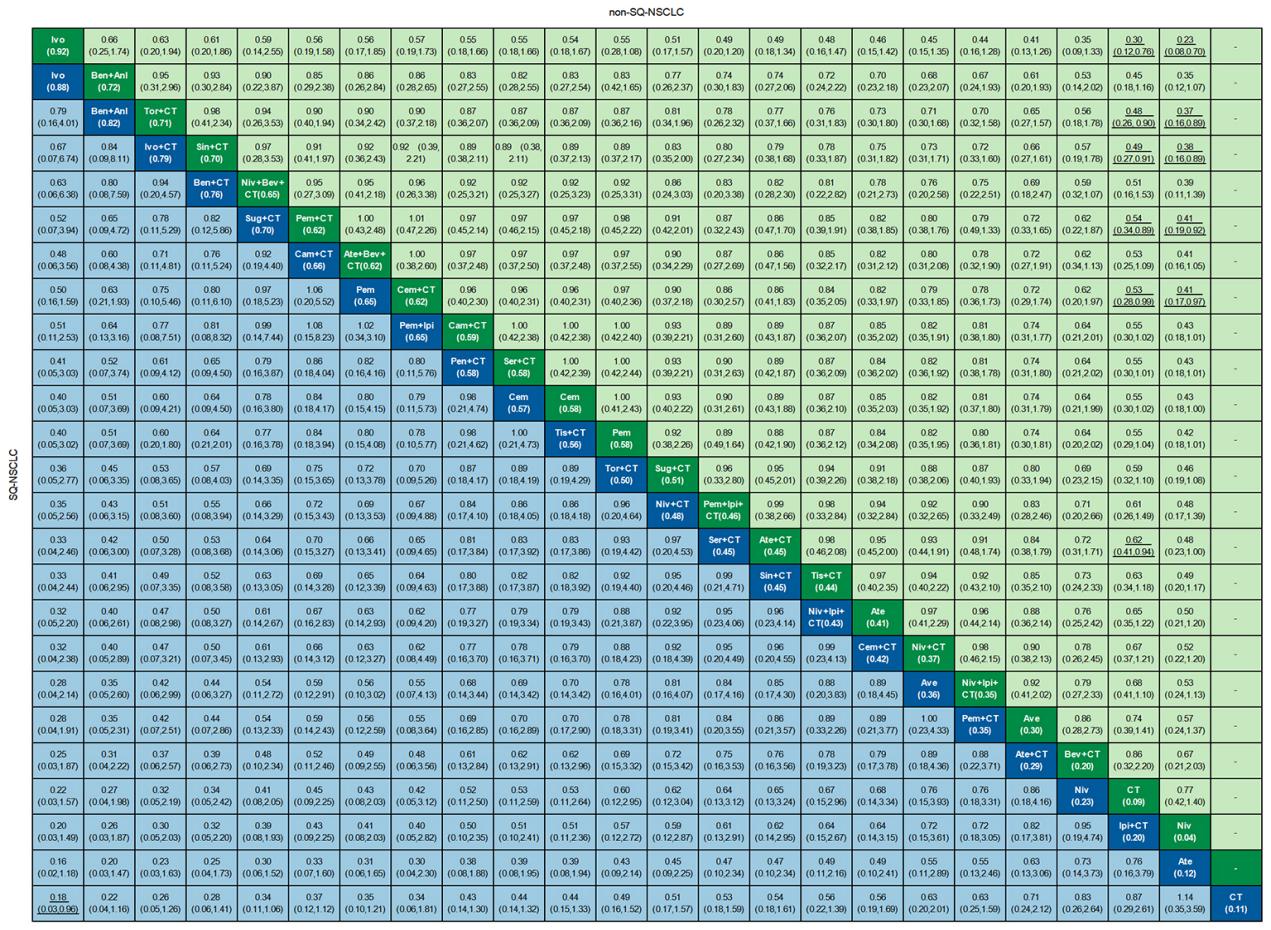


**Figure S10** Sensitivity analysis for PFS using a random‑effects model. Treatment regimens are presented in order of PFS ranking from high to low according to SUCRAs. Dark blue represents the regimens for SQ-NSCLC (SUCRA values), and dark green represents the regimens for non-SQ-NSCLC (SUCRA values). Data are HRs (95% CrIs) for column-defined treatment versus row-defined treatment for SQ-NSCLC (lower triangle) and row-defined treatment versus column-defined treatment for non-SQ-NSCLC (upper triangle). Significant results are underlined.

PFS, progression-free survival; SUCRA, surface under the cumulative ranking; HR, hazard ratio; CI, confidence interval; SQ-NSCLC, squamous non-small cell lung cancer; Anl, anlotinib; Ate, atezolizumab; Ave, avelumab; Ben, benmelstobart; Bev, bevacizumab; Cam, camrelizumab; Cem, cemiplimab; Dur, durvalumab; Ipi, ipilimumab; Ivo, ivonescimab; Niv, nivolumab; Pem, pembrolizumab; Pen, penpulimab; Ser, serplulimab; Sin, sintilimab; Sug, sugemalimab; Tis, tislelizumab; Tor, toripalimab; Tre, tremelimumab; CT, chemotherapy.

**
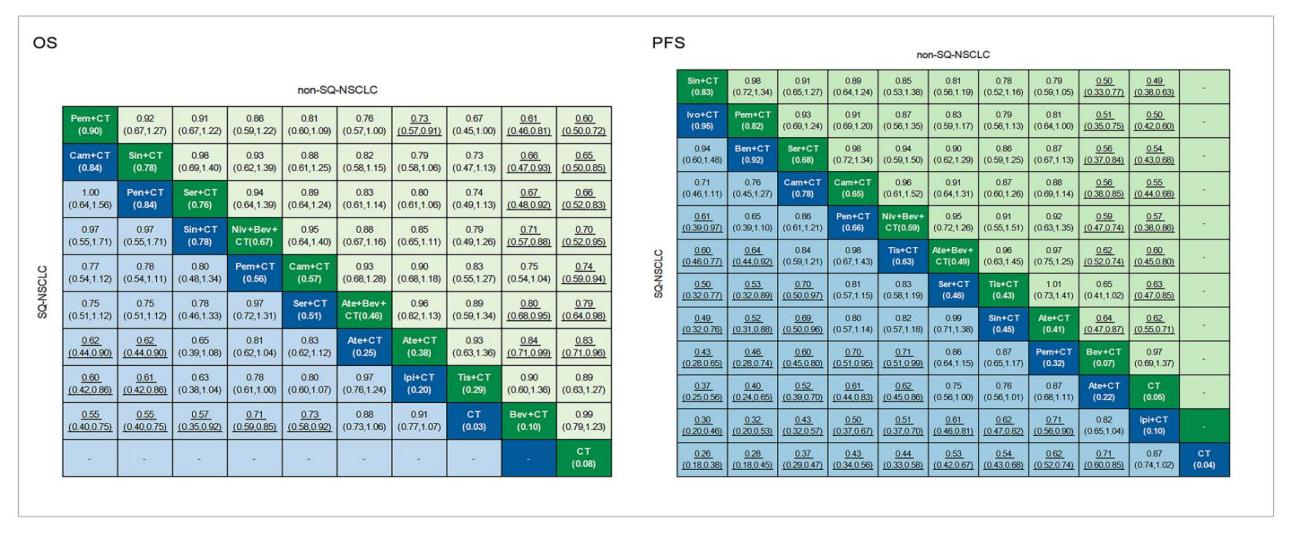
**

**Figure S11** Sensitivity analysis by restricting trials conducted exclusively in SQ-NSCLC or non-SQ-NSCLC. Treatment regimens are presented in order of OS or PFS ranking from high to low according to SUCRAs. Dark blue represents the regimens for SQ-NSCLC (SUCRA values), and dark green represents the regimens for non-SQ-NSCLC (SUCRA values). Data are HRs (95% CIs) for column-defined treatment versus row-defined treatment for SQ-NSCLC (lower triangle) and row-defined treatment versus column-defined treatment for non-SQ-NSCLC (upper triangle). Significant results are underlined.

OS, overall survival; PFS, progression-free survival; SUCRA, surface under the cumulative ranking; HR, hazard ratio; CI, confidence interval; SQ-NSCLC, squamous non-small cell lung cancer; Ate, atezolizumab; Ben, benmelstobart; Bev, bevacizumab; Cam, camrelizumab; Ipi, ipilimumab; Ivo, ivonescimab; Pem, pembrolizumab; Pen, penpulimab; Ser, serplulimab; Sin, sintilimab; Tis, tislelizumab; CT, chemotherapy.

**
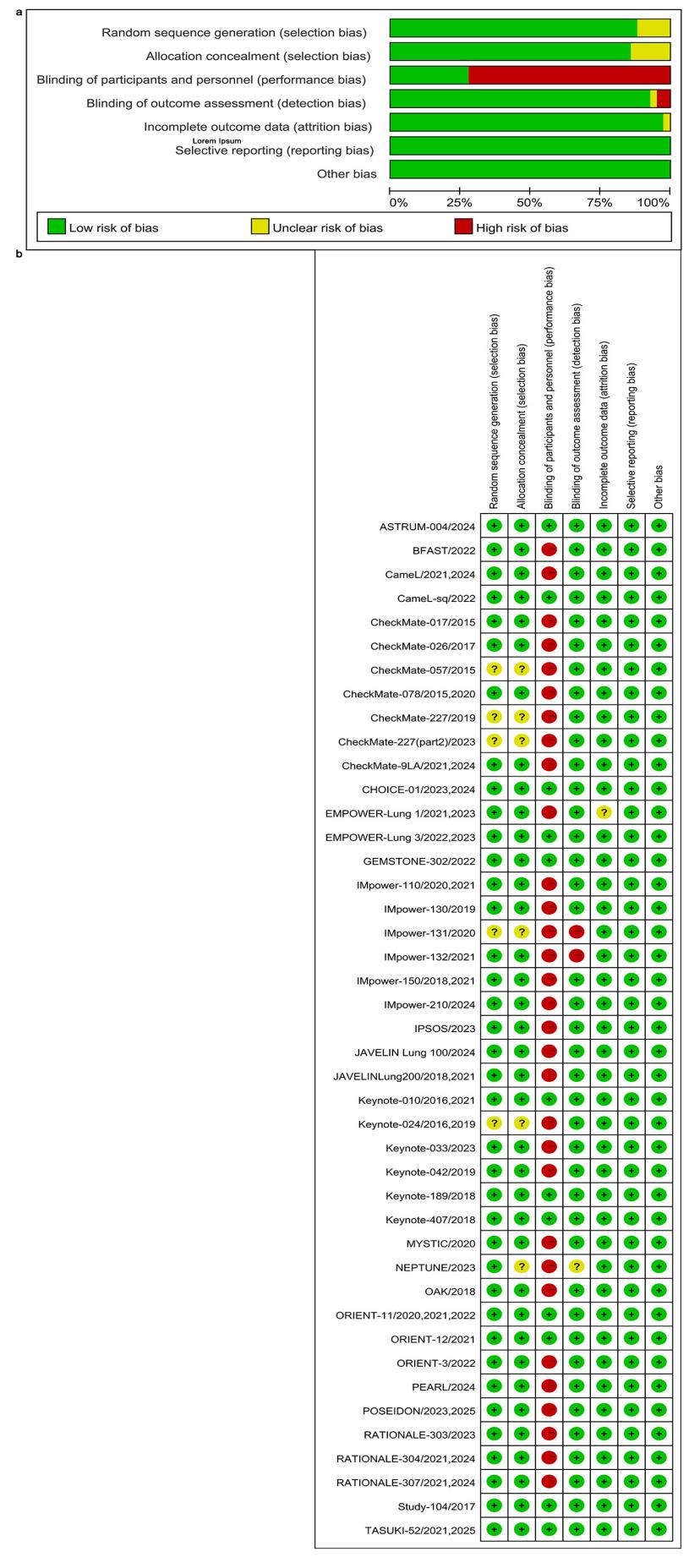
**

**Figure S12** Assessment of risk of bias. a: Methodological quality graph: authors’ judgment about each methodological quality item presented as percentages across all included studies; b: Methodological quality summary: authors’ judgment about each methodological quality item for each included study, “+” low risk of bias; “?” unclear risk of bias; “-” high risk of bias. RCTs, randomized controlled trials.
